# What Is the Link Between Attention-Deficit/Hyperactivity Disorder and Sleep Disturbance? A Multimodal Examination of Longitudinal Relationships and Brain Structure Using Large-Scale Population-Based Cohorts

**DOI:** 10.1016/j.biopsych.2020.03.010

**Published:** 2020-09-15

**Authors:** Chun Shen, Qiang Luo, Samuel R. Chamberlain, Sarah Morgan, Rafael Romero-Garcia, Jingnan Du, Xingzhong Zhao, Évelyne Touchette, Jacques Montplaisir, Frank Vitaro, Michel Boivin, Richard E. Tremblay, Xing-Ming Zhao, Philippe Robaey, Jianfeng Feng, Barbara J. Sahakian

**Affiliations:** a Institute of Science and Technology for Brain-Inspired Intelligence, MOE Key Laboratory of Computational Neuroscience and Brain-Inspired Intelligence, Fudan University, Shanghai, China; b State Key Laboratory of Medical Neurobiology and MOE Frontiers Center for Brain Science, Institutes of Brain Science and Human Phenome Institute, Fudan University, Shanghai, China; c Collaborative Innovation Center for Brain Science, Fudan University, Shanghai, China; d School of Mathematical Sciences, Fudan University, Shanghai, China; e Behavioural and Clinical Neuroscience Institute, Department of Psychology, University of Cambridge, Cambridge, United Kingdom; f Department of Psychiatry, University of Cambridge, Cambridge, United Kingdom; g Alan Turing Institute, London, United Kingdom; h Department of Psychoeducation, Université du Québec à Trois-Rivières, Trois-Rivières, Québec, Canada; i Department of Psychiatry, Université de Montréal, Montréal, Québec, Canada; j Department of Pediatrics and Psychology, Université de Montréal, Montréal, Québec, Canada; k School of Psychoeducation, Université de Montréal, Montréal, Québec, Canada; l School of Psychology, Université Laval, Québec City, Québec, Canada; m Department of Psychiatry, University of Ottawa, Ottawa, Ontario, Canada; n Children’s Hospital of Eastern Ontario, Ottawa, Ontario, Canada; o School of Public Health, Physiotherapy and Sports Science, University College Dublin, Dublin, Ireland; p Department of Computer Science, University of Warwick, Coventry, United Kingdom; q Shanghai Research Center for Brain Science and Brain-Inspired Intelligence, Institute of Brain-Intelligence Technology, Zhangjiang Laboratory, Shanghai, China; r Center for Advanced Research in Sleep Medicine, CIUSSS-NIM, Montréal, Québec, Canada

**Keywords:** ADHD, Development, Dyssomnia, Longitudinal study, Neurodevelopmental, Parasomnia

## Abstract

**Background:**

Attention-deficit/hyperactivity disorder (ADHD) comorbid with sleep disturbances can produce profound disruption in daily life and negatively impact quality of life of both the child and the family. However, the temporal relationship between ADHD and sleep impairment is unclear, as are underlying common brain mechanisms.

**Methods:**

This study used data from the Quebec Longitudinal Study of Child Development (*n* = 1601, 52% female) and the Adolescent Brain Cognitive Development Study (*n* = 3515, 48% female). Longitudinal relationships between symptoms were examined using cross-lagged panel models. Gray matter volume neural correlates were identified using linear regression. The transcriptomic signature of the identified brain-ADHD-sleep relationship was characterized by gene enrichment analysis. Confounding factors, such as stimulant drugs for ADHD and socioeconomic status, were controlled for.

**Results:**

ADHD symptoms contributed to sleep disturbances at one or more subsequent time points in both cohorts. Lower gray matter volumes in the middle frontal gyrus and inferior frontal gyrus, amygdala, striatum, and insula were associated with both ADHD symptoms and sleep disturbances. ADHD symptoms significantly mediated the link between these structural brain abnormalities and sleep dysregulation, and genes were differentially expressed in the implicated brain regions, including those involved in neurotransmission and circadian entrainment.

**Conclusions:**

This study indicates that ADHD symptoms and sleep disturbances have common neural correlates, including structural changes of the ventral attention system and frontostriatal circuitry. Leveraging data from large datasets, these results offer new mechanistic insights into this clinically important relationship between ADHD and sleep impairment, with potential implications for neurobiological models and future therapeutic directions.

Attention-deficit/hyperactivity disorder (ADHD) is the most prevalent neurodevelopmental disorder in children and persists into adulthood in 50% to 66% of cases (1, 2, 3). Sleep disturbances, composed of two main classifications (i.e., dyssomnia and parasomnia) (4), are extremely common, occurring in 25% to 55% of children with ADHD (5), and are associated with poor outcomes (6, 7, 8). Left untreated, such symptoms lead to untoward functional consequences in young people, including worse academic outcomes (9, 10, 11). Both ADHD symptoms and sleep problems can produce profound disruption in daily life and impact negatively quality of life of both the child and the family (12, 13, 14). ADHD symptoms such as hyperactivity can lead to longer sleep onset latency, more night awakening, and lower sleep quality (15), but disrupted sleep can also impair daytime attention, overlapping with the core symptoms of ADHD (16). Thus, these two conditions could mutually exacerbate each other (17). This complex relationship is of particular concern during childhood and adolescence when brain structures and associated functions are undergoing significant developmental changes (18, 19, 20). Both conditions have separately been linked to aberrant brain development (21, 22, 23, 24, 25). Therefore, greater knowledge of the temporal and neurobiological relationships between these two conditions is of considerable clinical and public health importance (26).

At the behavioral level, studies have reported associations between ADHD symptoms and sleep disturbance (27, 28, 29, 30), although most of this research has been cross-sectional. Furthermore, these studies often did not control for stimulant medication or examine brain substrates. At the neuroanatomical level, a wide range of brain abnormalities have been associated with ADHD (31), including structural abnormalities in both subcortical and cortical regions (32,33). In studies not considering ADHD symptoms, neuroanatomical correlates of sleep disturbances in patients with insomnia have also been identified in overlapping corticostriatal circuitry (34, 35, 36, 37). These findings often varied across studies, owing to both the heterogeneous nature of these conditions and methodological inconsistencies (e.g., age group recruited, symptom measurements used) (38,39). Nevertheless, one hypothesis is that delayed development of the cognitive control system (frontostriatal circuit) coupled with lower daytime arousal, implicating the salience/ventral attention pathway, contributes to these problems (40, 41, 42, 43, 44). While it has been hypothesized that common structural abnormalities of the frontostriatal and salience/ventral attention networks contribute to both conditions, this hypothesis has yet to be tested (45).

At the molecular level, ADHD has been associated with neurotransmitter systems, especially dopamine (46, 47, 48) and norepinephrine (49, 50, 51), which also play an important role in sleep regulation (52, 53, 54). For example, the locus coeruleus noradrenergic system, with widely projecting noradrenergic axons from brainstem to the central nervous system (e.g., hippocampus and neocortex), has been implicated in both attention and arousal (55). Dysregulation of this system has been hypothesized to be involved in not only sleep disturbances, but also the pathophysiology of ADHD (56). Therefore, investigating the interplay between sleep disturbances and ADHD symptoms may be particularly informative to shed new light on this hypothesis.

To our knowledge, no study to date using large-scale datasets has identified common brain abnormalities in both ADHD and sleep disturbance. Therefore, the objectives of the present study were 1) to uncover the temporal relationship between ADHD symptoms and sleep disturbances, 2) to identify the common neuroanatomical association shared between both symptom types, 3) to quantify the extent to which the identified neuroanatomical association was mediated through ADHD, and 4) to examine the gene expressions of which biological processes or functional pathways are associated with the mediation effect. To achieve these goals, we used 3 datasets: a longitudinal cohort of child development (*n* = 1601), a neuroimaging cohort with a longitudinal design (*n* = 3515), and an “all genes, all structures” gene expression survey in human brains (3702 samples with >62,000 gene probes per profile). We hypothesized that ADHD symptoms would contribute to subsequent sleep disturbances, that reduced gray matter volume (GMV) of frontostriatal and salience/ventral attention pathways would be common to both conditions, and that brain gene expression regulating the neurochemical systems above (dopamine, norepinephrine) would be related to mediating effects between brain structure and symptoms. Specifically, we postulated mechanistically that changes in brain structure and gene expression would contribute to ADHD, which in turn would contribute to sleep disturbance.

## Methods and Materials

### Participants and Behavioral Measures

We used data from the Quebec Longitudinal Study of Child Development (QLSCD) (57) as the discovery dataset for longitudinal analysis. Participants with at least one observation of ADHD symptoms or sleep disturbances at ages 7, 8, 10, 12, and 13 years were included in the present study (*n* = 1601). ADHD symptoms (total score, hyperactivity-impulsivity score, and inattention score) were measured using the teacher-rated Social Behavior Questionnaire (58). Sleep disturbances were assessed by 7 questions, which were answered by the mother (i.e., daytime sleepiness, sleep talking, sleep walking, night terror, nightmare, bruxism, and enuresis). The protocol of QLSCD was approved by the Quebec Institute of Statistics (Quebec City, Quebec, Canada) and the St-Justine Hospital Research Center (Montreal, Quebec, Canada) ethics committees. Written informed consent was obtained from all the participating families at each assessment. This cohort focused on behavioral measures and not brain imaging (Method S1 in Supplement 1).

The Adolescent Brain Cognitive Development (ABCD) Study is tracking the brain development and health of more than 10,000 children 9 to 11 years of age from 21 centers throughout the United States (https://abcdstudy.org). These centers obtained full written informed consent of parents and assent of all children, and research procedures and ethical guidelines were followed in accordance with the institutional review boards. We used data from 3515 subjects for whom both complete behavioral and magnetic resonance imaging (MRI) data were available at baseline; 3076 of these subjects had 1-year follow-up data available (Method S2 in Supplement 1). ADHD symptoms were measured using the parent-reported DSM-Oriented Attention Problem Scale of the Child Behavior Checklist (59). Sleep disturbances rated using the parent Sleep Disturbance Scale for Children (60) were further summarized into 2 dimensions: dyssomnias (disorders of initiating and maintaining sleep, sleep breathing disorders, and disorders of excessive somnolence) and parasomnias (disorders of arousal, sleep-wake transition disorders, and sleep hyperhidrosis) (61).

### Structural MRI Data

In the ABCD Study, 3-dimensional T1-weighted images were collected using 3T scanners at 21 data collecting sites. The detailed MRI acquisition protocol is described elsewhere (62). We obtained minimally preprocessed MRI data using the ABCD Pipeline (https://abcdstudy.org/scientists-protocol.html), and voxel-based morphometry analysis was conducted using a Computational Anatomy Toolbox (CAT12) (http://dbm.neuro.uni-jena.de/cat) and SPM12 (http://www.fil.ion.ucl.ac.uk/spm). Briefly, images were segmented into gray matter (GM), white matter, and cerebrospinal fluid based on tissue probability maps for ages 9 to 11 produced by the TOM8 Toolbox (https://irc.cchmc.org/software/tom.php). Next, images were normalized to the Montreal Neurological Institute space using the DARTEL toolbox and Geodesic Shooting approach. The registered GM images were multiplied with the Jacobian determinants derived from the spatial normalization and then smoothed with an 8-mm full width at half maximum Gaussian kernel with the resulting voxel size 1.5 mm^3^. Finally, we calculated the mean image of all these smoothed GM images and focused our subsequent analyses within a mask of GM by retaining only those voxels with more than 10% GM tissue.

### Transcriptomic Data

We used the transcriptomic data from 6 neurotypical adult brains in the Allen Human Brain Atlas (AHBA) (https://human.brain-map.org) (63). Because right hemisphere data were available for only 2 of the 6 donors in AHBA, we used samples in the left hemisphere only. We followed the preprocessing pipeline recommended by Arnatkevic Iūtė *et al.* (64), including probe-to-gene re-annotation, intensity-based data filtering, probe selection by mean, separating tissue samples into subcortical and cortical regions based on the Harvard-Oxford atlas (65), and within-donor normalization, finally resulting in 15,408 unique genes (Method S3 in Supplement 1).

### Statistical Analysis

#### Cross-Lagged Panel Analysis

In QLSCD, the longitudinal associations between ADHD total score and sleep disturbance were examined using a random-intercepts cross-lagged panel model (RI-CLPM) (66). Compared with the traditional CLPM, RI-CLPM requires at least 3 data waves and more closely approximates causal inference by separating the within-person process from stable between-person differences through the inclusion of random intercepts (67). We followed the two analytical steps in Madigan *et al.* (68). First, the standard RI-CLPM was estimated; then we examined the contribution of covariates (i.e., sex, socioeconomic status, and ADHD medication at age 7) to the between-person factors. We also performed RI-CLPMs between sleep and ADHD symptom dimensions (i.e., hyperactivity-impulsivity and inattention). We conducted a false discovery rate (FDR) correction for the 16 between-wave associations examined (i.e., 8 autoregressive and 8 cross-lagged paths).

To provide more supporting evidence using an independent cohort, we conducted traditional CLPMs for ADHD symptoms and each of the 3 sleep disturbance scores (i.e., total score, dyssomnia, and parasomnia) in the ABCD Study. We controlled for several stable variables (i.e., sex, race, and site) and time-variant parameters (i.e., ADHD medication, household income, educational level of parents, body mass index, and puberty) in these models. Accounting for the family relatedness (i.e., the records of single, sibling, twin, and triplet provided in a questionnaire as well as the kinship reconstructed from the genetic data) (Method S4 in Supplement 1; the code is available at the following link: https://github.com/qluo2018/FamilyPermutationABCD), the statistical significance, denoted by *p*_*perm*_, was established by conducting 5000 times multilevel block permutations (69). We compared the strength between the sleep→ADHD path and the ADHD→sleep path by the Wald test. To test whether the findings were robust across data collection sites, we conducted a meta-analysis of the significant cross-lagged coefficient identified above (Method S5 in Supplement 1). The model parameters were estimated by the full information maximum likelihood method (70), and the model fit was interpreted using common thresholds of good fit (71).

#### Whole-Brain and Voxelwise Analysis (ABCD Cohort)

A linear regression model was conducted to investigate the relationship between GMVs and ADHD symptoms at baseline, using age, sex, handedness, race, puberty, body mass index, site, household income, parental education, head motion, and total intracranial volume as covariates of no interest. We conducted a multilevel block permutation-based cluster-level correction (5000 times) for multiple comparisons in the neuroimaging analysis (Method S4 in Supplement 1) (69,72,73). At voxel level, we used a 2-sided test with a significance level of α = .001, whereas at cluster level, we used a permutation-based familywise error correction with α = .05. Similarly, we examined the GMV correlates with the total sleep disturbance score (familywise error correction *p* < .05) and tested such correlations for 2 dimensions (i.e., dyssomnia and parasomnia, familywise error correction *p* < .025). Significant overlapping GMVs of ADHD and sleep were defined on the basis of a cluster having more than 217 voxels falling into the 90% confidence interval (CI) of the smoothing kernel voxels (74).

#### Mediation Analysis

As the directional association between ADHD symptoms and sleep disturbances was determined by the CLPM, we assessed the mediation effect of ADHD on the association between sleep and the overlapping clusters identified above. The analyses were performed using the mediation toolbox developed by Wager *et al.* (75) (https://github.com/canlab/MediationToolbox) with 10,000 bootstraps.

Furthermore, we conducted a whole-brain and voxelwise exploratory analysis of this mediation effect with 3000 bootstraps at each voxel and FDR correction among all voxels. We additionally required a significant GMV-sleep association (*p* < .005, 2-sided, uncorrected). The unthresholded bootstrap-based *t* map of the mediation effect was further used in the following analyses.

#### Transcriptomic Analysis

We used partial least square (PLS) regression to relate the mediation effect to the gene expression data in AHBA (76, 77, 78, 79). The response variable was an *n* × 1 matrix, which was calculated by the average *t* value of a spherical region of interest (ROI) (*r* = 4 mm) centered by the Montreal Neurological Institute coordinates of each gene expression sampling site (80). The predictor variable was an *n* (number of tissue samples) × 15,408 (number of genes) matrix. AHBA provided 182 tissue samples in left subcortical regions and 784 tissue samples in left cortical regions. Using a permutation test (5000 times), we selected the PLS components that explained more variance of the mediation effect than could be accounted for by chance (76,79). The first PLS component was the linear combination of the weighted gene expression scores, maximizing the covariance between the expression profile and the mediation profile in the brain. A *z* score was calculated for each weight in a PLS component as the ratio between each weight estimation and standard error given by 5000 bootstraps. Therefore, the genes could be ranked by their normalized contributions to the PLS component. We adapted the codes for PLS provided by others (76,79). Leave-one-out cross-validation (i.e., repeating the analysis by leaving 1 donor out at a time) was used to test the influence of individual donors on the results, and PLS analyses using 6-mm regions of interest or a refined brain atlas (78,81) were also performed and compared to ensure that the findings were not dependent on a particular definition of region of interest size.

We used GSEAPreranked (version 6.0.12) (82) with default settings to identify sets of genes associated with Gene Ontology terms of biological processes and Kyoto Encyclopedia of Genes and Genomes pathways. The 2 lists of genes (*n* = 15,408) for subcortical and cortical regions were ranked by *z* score and passed to GSEAPreranked. From the top positively and negatively correlated genes in each list, we obtained separate sets of enriched gene sets (S+ and S−, respectively). To demonstrate the robustness of the findings, we also applied a more stringent threshold (i.e., top 1% and bottom 1% genes based on *z* score) to identify significant enrichments using DAVID 6.8 (83). Gene sets were considered significantly enriched with FDR *q* values < .05.

## Results

### Demographics

From QLSCD, 1601 participants (829 [52%] female) with behavioral measurements that were longitudinally collected at 7, 8, 10, 12, and 13 years of age were entered into the current study (Table 1). From the ABCD Study, 3515 participants (1664 [48%] female, 10 ± 0.61 years old) who had both complete MRI data and behavioral measurements at baseline were used in the current study, of whom 3076 had complete behavioral assessments at a 1-year follow-up (11.03 ± 0.63 years old) (Table 2).

**Table 1:** Characteristics of the Study Population in the Quebec Longitudinal Study of Child Development (QLSCD)

	Wave 1		Wave 2		Wave 3		Wave 4		Wave 5	
	Mean (± SD)	*n*	Mean (± SD)	*n*	Mean (± SD)	*n*	Mean (± SD)	*n*	Mean (± SD)	*n*
Sex, Male, *n* (%)	772 (48.2%)	1601								
Age, Years	7.15 (± 0.25)	1468	8.15 (± 0.26)	1421	10.15 (± 0.26)	1295	12.14 (± 0.25)	1353	13.14 (± 0.26)	1252
ADHD Medication, *n* (%)	56 (3.8%)	1468								
SES	−0.01 (± 1.00)	1467								
ADHD Total Score	2.64 (± 2.56)	1303	2.55 (± 2.43)	1267	2.31 (± 2.37)	986	2.13 (± 2.21)	1004	2.52 (± 2.56)	992
H-I Score	2.05 (± 2.52)	1302	1.90 (± 2.40)	1266	1.62 (± 2.33)	986	1.39 (± 2.09)	999	1.48 (± 2.39)	937
IN Score	3.79 (± 3.39)	1303	3.77 (± 3.32)	1281	3.63 (± 3.27)	987	3.57 (± 3.32)	1004	3.76 (± 3.25)	1023
Sleep Disturbance	0.23 (± 0.25)	1601	0.26 (± 0.25)	1601	0.32 (± 0.24)	1062	0.29 (± 0.22)	1188	0.28 (0.22)	832

Descriptive statistics are reported as mean (± SD) unless noted otherwise.

ADHD, attention-deficit/hyperactivity disorder; H-I, hyperactivity-impulsivity; IN, inattention; SES, socioeconomic status.

**Table 2:** Characteristics of the Study Population in the Adolescent Brain Cognitive Development (ABCD) Study

	Baseline (*n* = 3515)	Follow-up (*n* = 3076)
Age, Years	10.00 (± 0.61)	11.03 (± 0.63)
Sex, Male, *n* (%)	1851 (52.7%)	1610 (52.3%)
Puberty	1.56 (± 0.46)	1.79 (± 0.59)
BMI	18.59 (± 4.00)	19.45 (± 4.52)
Parental Education	17.01 (± 2.47)	17.16 (± 2.33)
Household Income	7.52 (± 2.19)	7.76 (± 2.06)
Race, *n* (%)		
White	2554 (72.7%)	2307 (75%)
Black/African American	327 (9.3%)	244 (7.9%)
Asian	182 (5.2%)	155 (5%)
Other	452 (12.9%)	370 (12%)
Family Relationship, *n* (%)^a^		
Single	2680 (76.2%)	2346 (76.3%)
Sibling	196 (5.6%)	166 (5.4%)
Twin	630 (17.9%)	558 (18.1%)
Triplet	9 (0.3%)	6 (0.2%)
ADHD Symptoms	2.54 (± 2.91)	2.36 (± 2.86)
ADHD Medication, *n* (%)		
No medication	3204 (91.2%)	2820 (91.7%)
Stimulant only	233 (6.6%)	193 (6.3%)
Nonstimulant only	31 (0.9%)	27 (0.9%)
Stimulant + nonstimulant	46 (1.3%)	36 (1.2%)
Sleep Disturbance		
Total score	36.24 (± 7.87)	36.41 (± 7.75)
Dyssomnia	22.19 (± 5.49)	22.66 (± 5.67)
Parasomnia	14.05 (± 3.58)	13.75 (± 3.32)

Descriptive statistics are reported as mean (± SD) unless noted otherwise.

ADHD, attention-deficit/hyperactivity disorder; BMI, body mass index.

a Provided by a questionnaire (“acspsw02”) from ABCD dataset.

### ADHD Symptoms Contributed to Sleep Disturbance in School-Aged Children

**Figure 1:**
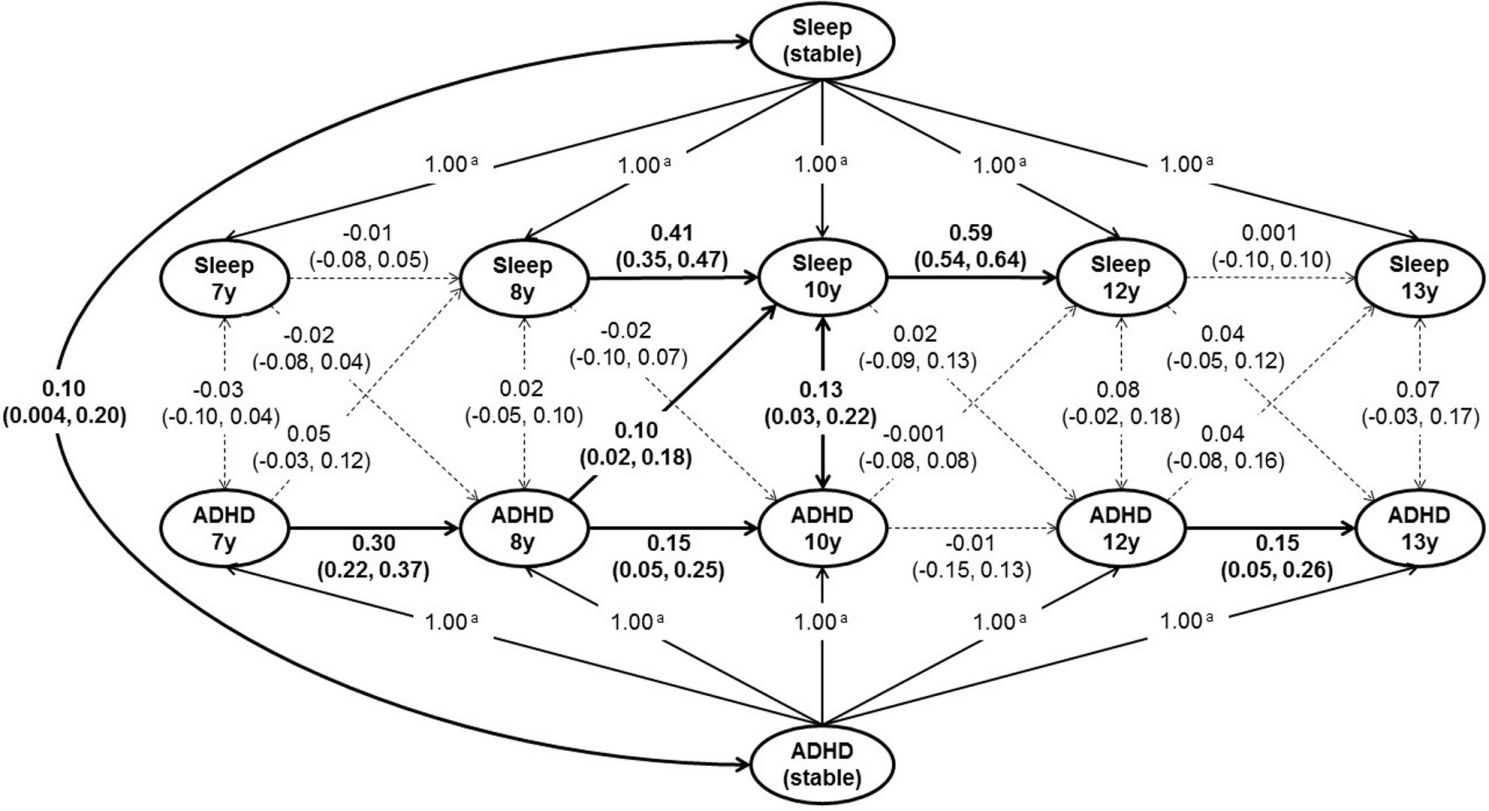
Cross-lagged analysis between attention-deficit/hyperactivity disorder (ADHD) and sleep disturbance in the Quebec Longitudinal Study of Child Development. Random-intercepts cross-lagged panel model of ADHD total score and sleep disturbance from ages 7 to 13 years in the Quebec Longitudinal Study of Child Development (*n* = 1601). Standardized estimates (95% confidence intervals) are presented. Solid lines represent statistical significance (*p* < .05), whereas dashed lines represent nonsignificance (*p* > .05). Model fit: root mean square error of approximation = 0.04; comparative fit index = 0.98; Tucker-Lewis index = 0.96; standardized root mean square residual = 0.04. ^*a*^Pathways constrained to 1.00 to isolate between-person factor.

To test the directionality, if any, between ADHD symptoms and sleep disturbances, we conducted a longitudinal analysis using the QLSCD cohort. We found a between-person and time-invariant association between ADHD total score and sleep disturbance (β = .10, 95% CI [0.004, 0.20]). In the within-person and dynamic component of the model, we found that higher ADHD total score at age 8 years was associated with worse sleep disturbance at age 10 (noted as ADHD 8y→sleep 10y) (β = .10, 95% CI [0.02, 0.18], FDR *q* < .05) (Table S1 in Supplement 1; Figure 1). Considering the covariates (i.e., sex, socioeconomic status, and ADHD medication), ADHD 8y→sleep 10y remained significant (β = .11, 95% CI [0.03, 0.19], FDR *q* = .02) (Figure S1 in Supplement 1). Both additional analyses using the participants without ADHD medication only (*n* = 1095; β = .09, 95% CI [0.004, 0.18]) and using the participants who only had data at both ages 8 and 10 years (*n* = 1263; β = .09, 95% CI [0.01, 0.18]) (Result S1 in Supplement 1) confirmed the significance of ADHD 8y→sleep 10y. Similar results held for ADHD subscales (i.e., hyperactivity-impulsivity symptom and inattention symptom) (Figures S2 and S3 in Supplement 1).

Using the ABCD cohort, we found more supporting evidence that ADHD symptoms at age 10 were also associated with sleep disturbances at a 1-year follow-up (sleep total score: β = .09, 95% CI [0.06, 0.13], *p*_*perm*_ < .001) (Figure S4A in Supplement 1), and the meta-analysis across all data collection sites showed that this association was also robust (meta-β = .09, 95% CI [0.04, 0.13]). In contrast, although the path coefficient of the opposite direction was significant in this large cohort, its effect size was weaker (*p* < .005 by Wald test) and not robust (Figure S5A, B in Supplement 1). Again, both additional analyses using the participants without ADHD medication only (*n* = 2902; β = .04, 95% CI [0.01, 0.08]) and using the participants who were diagnosed with ADHD at baseline (*n* = 281; β = .10, 95% CI [0.01, 0.20]) (Result S2 in Supplement 1) confirmed the significance of ADHD→sleep. Similar results held for sleep subscales (i.e., dyssomnia and parasomnia) (Figures S4 and S5 in Supplement 1).

### Shared Neural Correlates Between ADHD Symptoms and Sleep Disturbances

**Figure 2:**
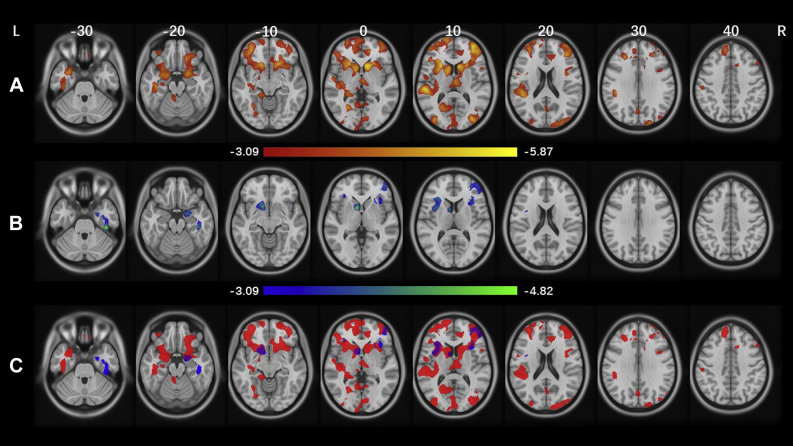
Significant brain clusters associated with attention-deficit/hyperactivity disorder (ADHD) symptoms and dyssomnia in the Adolescent Brain Cognitive Development Study at baseline. Multiple comparison correction includes voxel-level *p* < .001 and cluster-level familywise error correction *p* < .05 for ADHD and *p* < .025 for dyssomnia estimated by a multilevel block permutation accounting for family relatedness. The color bar represents *t* value. Age, sex, handedness, race, puberty, body mass index, site, household income, parental education, head motion, and total intracranial volume were controlled for in all analyses. **(A)** Brain regions significantly associated with ADHD symptoms. **(B)** Brain regions significantly associated with dyssomnia. **(C)** Brain regions significantly associated with ADHD or dyssomnia. Red areas are associated with ADHD, blue areas are associated with dyssomnia, and purple areas are the overlapping regions. L, left; R, right.

To test the hypothesis that ADHD symptoms and sleep disturbances share common neural correlates, we conducted a neuroimaging analysis using the ABCD cohort at baseline. We found that ADHD symptoms were associated with lower GMV in 2 brain clusters (Figure 2A; Tables S2 and S3 in Supplement 1; Figures S6 and S7A in Supplement 1), while only the dyssomnia subscale was associated with lower GMV in 3 brain clusters (Figure 2B; Tables S3 and S4 in Supplement 1; Figure S7B in Supplement 1). Among these clusters, we found 3 overlapping areas, including in the bilateral insula, left caudate, and putamen (2762 voxels); in the right middle frontal gyrus and inferior frontal gyrus (2296 voxels); and in the right parahippocampus, hippocampus, and amygdala (419 voxels) (Figure 2C; Figure S7C in Supplement 1).

### Mediation Analysis: Identified Neural Correlates Contributed to Both ADHD Symptoms and Dyssomnia

**Figure 3:**
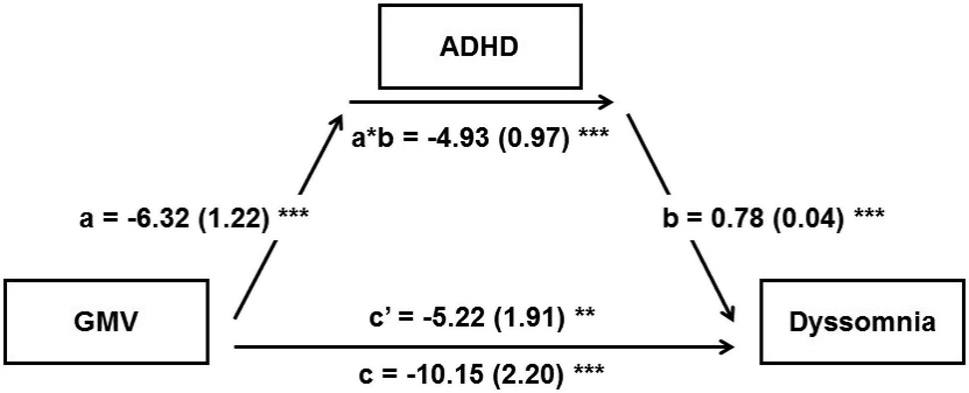
Associations of average overlapping gray matter volume (GMV), attention-deficit/hyperactivity disorder (ADHD) symptoms, and dyssomnia. Mediation model using the average overlapping GMV as the predictor, ADHD as the mediator, and dyssomnia as the dependent variable. Age, sex, handedness, race, puberty, body mass index, site, household income, parental education, head motion, and total intracranial volume were used as covariates of no interest. Path a measures the association between the predictor and the mediator; path b represents the effect of the mediator on the dependent variable while controlling for the predictor; path c measures the total relationship between the predictor and the dependent variable; path c′ measures the direct effect; the mediation effect is the product of path a and path b (a ∗ b). ∗∗*p* < .01, ∗∗∗*p* < .001.

Having identified that ADHD contributed to subsequent sleep problems in the longitudinal datasets, we were then interested in examining whether ADHD statistically contributed to anatomical brain changes linked with sleep problems cross-sectionally. Thus, we conducted a mediation analysis to quantify the brain→ADHD→sleep relationship. We found that 48.6% (95% CI [31.7%, 75.9%]) of the association between lower average GMV of the 3 overlapping areas identified above and higher dyssomnia was mediated by ADHD symptoms (the mediation effect of each area was between 45.2% and 49.7%) (Figure 3; Figure S8 in Supplement 1). Controlling for ADHD medication, this mediation effect remained significant (44.2%, 95% CI [27.3%, 77.2%]; path a ∗ b: −3.92, 95% CI [−5.64, −2.35]). The whole-brain exploratory analysis of the mediation effect identified a significant cluster that covered the overlapping areas identified above (Figure S9 in Supplement 1). We also confirmed that ADHD symptoms at baseline significantly mediated the association between the baseline GMV and the follow-up dyssomnia (Figure S10 in Supplement 1).

### Relationship Between the Mediation Effect and Brain Gene-Expression Profiles

Given that the strength of the brain→ADHD→sleep relationship had a heterogeneous spatial distribution among different brain areas, we further conducted a transcriptomic analysis to identify its association with the gene expressions in brain tissues. In the model for the subcortical regions, only the first PLS component was significant (i.e., explained 30% of the variance of the mediation effect, *p* < .001 by permutation) (additional details and validations in Results S3–S6 in Supplement 1). After the FDR correction, the S− gene sets in subcortical regions were enriched in 104 relevant biological processes (Table S7 in Supplement 2) and 26 relevant Kyoto Encyclopedia of Genes and Genomes pathways (Table S8 in Supplement 2). Enriched biological processes included chemical synaptic transmission, while enriched Kyoto Encyclopedia of Genes and Genomes pathways included circadian entrainment and dopaminergic synapse. These findings were also enriched in the top 1% gene set (Table S6 in Supplement 1). More enrichment findings of the S+ gene sets and the cortical regions are listed in Tables S9–S14 in Supplement 2.

## Discussion

This study demonstrated that the cross-lagged association of ADHD at baseline with sleep disturbance at follow-up was stronger than the cross-lagged association in the opposite direction. Neuroimaging analysis revealed that smaller volumes, mainly in the cognitive control system and the salience/ventral attention system, constituted a common neurobiological substrate linking both ADHD and sleep disturbance. Among the subcortical structures, we identified a number of genes with higher expression levels in those brain areas where a greater proportion of the brain-sleep association was mediated by ADHD. These genes included those playing key roles in dopamine signaling and in the circadian cycle. The findings are in keeping with our hypothesis that changes in brain structure and gene expression contribute to ADHD, which in turn leads to sleep disturbance.

To our knowledge, this is the first study using RI-CLPM to address the longitudinal associations between ADHD with sleep disturbances. RI-CLPM offers potential advantages over other statistical approaches, such as providing closer approximation of causal inference (67). Although cross-sectional associations between ADHD and sleep disturbances have been reported, little is known about the longitudinal relationships. Most longitudinal studies examined unidirectional associations only, such as early sleep patterns predicting later ADHD symptoms (84,85) or childhood ADHD symptoms being associated with adulthood sleep quality (86). In the QLSCD cohort, we found that the strength of the ADHD→sleep relationship peaked between 8 and 10 years of age. However, this finding did not necessarily suggest an age-restricted relationship. For example, the ADHD symptoms at age 10 might indirectly influence the sleep disturbances at age 12 via elevating the sleep disturbances at age 10 (Figure 1).

We found that ADHD symptoms and dyssomnia were associated with common reductions of GM in the right frontal gyrus, bilateral insula, left striatum, right amygdala, and hippocampus. Structural abnormalities of these regions have previously been reported separately in ADHD (87, 88, 89) and dyssomnia (35,36,90, 91, 92). Our findings are in keeping with prior data, but crucially extend beyond it to identify common underpinnings of these 2 related pathologies by leveraging a large dataset (93). These neural regions play a cardinal role in high-level cognitive functions (frontostriatal circuitry) (94) and in the salience/ventral attention system (95).

Cognitive domains contingent on the frontostriatal circuitry are often impaired in ADHD (96) and in people with sleep disturbances (97). The striatum is connected to prefrontal cortex (98,99) and is particularly implicated in ADHD (100) as well as being important for sleep-wake regulation (101) and arousal (35). Disturbances in the maturation of such frontostriatal circuitry may contribute to cognitive problems often found in ADHD, such as difficulties in self-regulation (102), cognitive control (103), and reward processing (104). Notably, attention and arousal closely interact with each other (105), and their interaction has been hypothesized to be mainly located at the salience/ventral attention pathway. Our findings are in keeping with disruption of these pathways being associated with ADHD and sleep disturbances, particularly with regard to the right inferior frontal cortex, right temporoparietal junction, right middle frontal gyrus, and anterior insular cortex (105, 106, 107). Such ADHD-related brain abnormalities have been posited to reflect delayed development of frontostriatal circuitry underlying cognitive control (21,108).

Notably, our findings provide novel insights into neurobiological mechanisms contributing to the relationship between ADHD symptoms and sleep disturbances. Specifically, we demonstrated that lower GMV in key brain regions was associated with ADHD and dyssomnia and that 40% of this neuroanatomical association was mediated by the impact of ADHD on sleep. Furthermore, our enrichment analysis of this mediation effect highlighted several overlapping pathways (Figure S15 in Supplement 1), including the circadian entrainment pathway and neural signaling pathways (e.g., chemical synaptic transmission, dopaminergic synapse, glutamatergic synapse). Particularly, the neuroanatomical associations of sleep disturbances were mediated to a greater extent by ADHD symptoms in subcortical regions with higher gene expression levels of these pathways (Figure S16 in Supplement 1). Therefore, if a given ADHD treatment targets one of these pathways, it might reduce the ADHD component in sleep disturbances (109). However, approximately half of the brain-sleep association was not mediated by ADHD symptoms, suggesting that not all GMV reductions common to ADHD and dyssomnia stem from ADHD itself. This suggests that additional sleep management strategies are needed from a treatment perspective, even though treating ADHD itself may lead to sleep improvements, provided that such treatments do not have their own deleterious effects (110).

Our study has several limitations. Using 2 large longitudinal cohorts, we observed a significant temporal relationship between ADHD symptoms and subsequent sleep disturbances in school-aged children. This relationship was greatest between ages 8 and 10 years in QLSCD and between ages 10 and 11 years in the ABCD Study. There are several possible reasons for this cohort difference, including the following: 1) As these 2 cohorts were collected 10 years apart (i.e., the QLSCD children were born in 1997–1998, while the ABCD Study children were born in 2007–2008), the same chronological age may not reflect the same pubertal stage in these 2 cohorts. 2) Given the significant development during adolescence, it is also possible that a 1-year follow-up after 10 years of age can be different from a 2-year follow-up after the same age. It is possible that future longitudinal studies could directly investigate these points.

### Conclusions

Analysis of 2 large longitudinal cohorts combined with the largest neuroimaging cohort of school-aged children to date revealed a strong ADHD-driven effect on subsequent sleep disturbance and identified common neuroanatomical correlates of both ADHD symptoms and sleep disturbances. We found that ADHD substantially mediated common neuroanatomical changes linked with both problems, highlighting the need to develop precision treatment approaches that integrate multimodal approaches to mitigate both ADHD symptoms and sleep disturbances.

## Supplementary Material

Supplement 1

Key Resources Table
