# Supplementary material for: What Is the Link Between Attention-Deficit/Hyperactivity Disorder and Sleep Disturbance? A Multimodal Examination of Longitudinal Relationships and Brain Structure Using Large-Scale Population-Based Cohorts
Source: Biol Psychiatry. 2020 Sep 15;88(6):459–69. doi: 10.1016/j.biopsych.2020.03.010 (PMC7445427; doi:10.1016/j.biopsych.2020.03.010)
Supplement: Supplement 1 [file mmc1.pdf]

What Is the Link between Attention-Deficit/Hyperactivity Disorder and Sleep Disturbance? A Multimodal Examination of Longitudinal Relationships and Brain Structure Using Large-Scale Population-based Cohorts

Supplement 1

Method S1. Quebec Longitudinal Study of Child Development (QLSCD) 3
Method S2. The Adolescent Brain Cognitive Development (ABCD) Study 4
Method S3. Preprocessing of the AHBA data 6
Method S4. CLPM and MRI analysis considering the family relatedness based on genetic data 7
Method S5. Meta-analyses of path coefficients in each ABCD site.............................8
Result S1. RI-CLPM using the same participants at age 8 and 10 years.......................9
Result S2. CLPMs using children with ADHD in the ABCD cohort..........................10
Result S3. PLS results...11
Result S4. Leave-one-out validation of transcriptomic analysis 12
Result S5. Validation of transcriptomic analysis using 6mm ROIs............................14
Result S6. Validation of transcriptomic analysis in cortical regions using a refined brain atlas. 15
Table S1. Results of FDR correction of RI-CLPM in QLSCD. 16
Table S2. Significant GMVs associated with ADHD symptoms corrected for the family relatedness defined by the questionnaire. 17
Table S3. Significant GMVs associated with ADHD symptoms corrected for the family relatedness reconstructed from the genetic kinship. 18
Table S4. Significant GMVs associated with dyssomnia corrected for the family relatedness defined by the questionnaire. 19
Table S5. Significant GMVs associated with dyssomnia corrected for the family relatedness reconstructed from the genetic kinship. 20
Table S6. Significant biological processes and KEGG pathways of the top and bottom 1% genes in subcortical and cortical regions. 21
Figure S1. RI-CLPM of ADHD total score and sleep disturbance from ages 7 to 13 years in QLSCD with covariates...22

Figure S2. RI-CLPM of hyperactivity-impulsivity symptom and sleep disturbance from ages 7 to 13 years in QLSCD with covariates....23
Figure S3. RI-CLPM of inattention symptom and sleep disturbance from ages 7 to 13 years in QLSCD with covariates....24
Figure S4. CLPMs of ADHD symptoms and sleep disturbance from baseline to follow-up in the ABCD study....25
Figure S5. Forest plots of meta-analyses of path coefficients across 18 data collection sites in the ABCD cohort....27
Figure S6. 3-D view of significant GMVs associated with ADHD symptoms corrected for the family relatedness defined by the questionnaire....29
Figure S7. Significant brain clusters associated with ADHD symptoms and dyssomnia corrected for the family relatedness reconstructed from the genetic kinship....30
Figure S8. Mediation models of average GMV in 3 overlapping clusters separately. 31
Figure S9. Results of the exploratory whole-brain voxel-wise mediation analysis....32
Figure S10. Associations of the baseline average overlapping gray matter volume, baseline ADHD symptoms and follow-up dyssomnia....33
Figure S11. Subcortical tissue samples in AHBA used in partial least square regression....34
Figure S12. Subcortical gene expression data in principal component (PC) space....35
Figure S13. Cortical tissue samples in AHBA used in partial least square regression. 36
Figure S14. Cortical gene expression data in principal component (PC) space....37
Figure S15. Enrichment network of KEGG pathways of S- gene set in subcortical regions....38
Figure S16. Scatterplots of gene expression vs. t statistics of mediation effect....39
Supplemental References....40

Method S1. Quebec Longitudinal Study of Child Development (QLSCD)

The QLSCD surveyed a representative sample of single children born in 1997–98 in the province of Quebec, Canada, except for children living in Cree/Inuit territories, Indian reserves, and northern Quebec (1). All children were recruited through the Quebec Master Birth Registry via a stratified procedure based on living area and birth rate. Families were included if the pregnancy had lasted between 24 and 42 weeks, and the mother could speak French or English. The protocol of QLSCD was approved by the Quebec Institute of Statistics (Quebec City, Quebec, Canada) and the St-Justine Hospital Research Center (Montreal) ethics committees. Written informed consent was obtained from all the participating families at each assessment. The initial sample comprised of 2,120 children aged 3–8 months (mean age 5 months), and followed every one to two years. Attrition rate and additional cohort characteristics are presented on the QLSCD website (http://www.iamillbe.stat.gouv.qc.ca/default_an.htm).

Measures

ADHD

In school-aged waves (age 7, 8, 10, 12 and 13 years), teachers were invited to complete the Social Behavior Questionnaire (2), which has been used in a large number of children's social and psychological development studies and was shown to have good reliability and validity (3, 4). All items referred to the conditions in the past six months. Five items of hyperactivity-impulsivity (e.g. "could not sit still, was restless or hyperactive") and three items of inattention (e.g. "was inattentive") were rated on a three-point scale (0=never or not true, 1=sometimes or somewhat true, 2=often or very true). ADHD total score, hyperactivity-impulsivity score and inattention score were calculated; higher scores indicate the more severe symptoms.

Sleep disturbance

Sleep disturbance was measured by seven mother-rated questions (i.e., "in general, is your child sleepy during the day?", "does your child talk/walk/have nightmares/have night terrors/grind teeth/pee in sleep?") on a four-point scale (0=never, 1=sometimes, 2=often, 3=always). A composite score was calculated by the average of these seven items. A higher score indicates the more severe sleep problems.

Covariates

Family socioeconomic status (SES) at age 7 was measured on the basis of education level of parents, the prestige of parents' occupation, and household income. These five variables were standardized and then averaged to create the composite SES score (5). ADHD medication at age 7 was collected by a dichotomous question ('in the past 12 months, does your child take Ritalin or any other medication that threat hyperactivity or inattention on a regular basis?'), and reported by parents.

Method S2. The Adolescent Brain Cognitive Development (ABCD) Study

The ABCD study is a large-scale longitudinal study of children brain development and mental health. Over 10,000 children aged 9 to 11 years are planned to be recruited from 21 centers throughout the United States. The 21 centers obtained parents' full written informed consent and all children's assent, and research procedures and ethical guidelines were followed in accordance with the Institutional Review Boards (IRB). We used data from the Annual Curated Data Release 1.1, which were collected between September 1, 2016 and September 15, 2017 from the baseline visits of 4,521 participants. Finally, 3,515 subjects with completed behavioral measurements and qualified structural MRI data were involved in the present study. In addition, 3,076 of 3,515 subjects had completed 1-year-followup data from the Data Release 2.0 in March 2019.

Measures

ADHD

At baseline and 1-year follow-up visits, parents were asked to complete the Child Behavior Checklist (6) to assess the dimensional psychopathology of children. We used the DSM-Oriented Attention Problem Scale to represent ADHD symptoms, which was superior to the original Attention Problem Scale in the identification of ADHD participants (7). A higher score indicates more severe ADHD symptoms.

Sleep disturbance

The Sleep Disturbance Scale for Children is a 26-item 4-Likert questionnaire (8), rated by parents. In the scale, six factors which represents the most common areas of sleep disorders in childhood and adolescence can be calculated, and can further be summarized into two dimensions: dyssomnia included disorders of initiating and maintaining sleep, sleep breathing disorders and disorders of excessive somnolence; parasomnia included disorders of arousal, sleep-wake transition disorders and sleep hyperhidrosis. Therefore, we used three kinds of sleep disturbance scores (total score, dyssomnia and parasomnia) in the analyses.

Covariates

Pubertal status was measured by the parent Pubertal Development Scale (9), which is consisted of three common questions and two sex-specific questions for boys and girls. Body mass index (BMI) is calculated by the body weight divided by the square of the body height. Handedness was assessed by the youth Edinburgh Handedness Inventory Short Form (10), scaling from 1 to 3 (1=right handed, 2=left handed, 3=mixed handed), and transformed into two dummy variables in analyses. Medication was recorded by asking parents "Did your child take any medications in the past two weeks". ADHD medication was categorized into 3 groups: stimulant drugs (i.e. Amphetamine, Dextroamphetamine, Amfetamine, Dextroamfetamine, Dexamphetamine, Dyanavel, Evekeo, Adderall, Mydayis, Dexedrine, Dexmethylphenidate, Focalin,

Lisdexamfetamine, Lisdexamphetamine, Vyvanse, Methylphenidate, Aptensio, Concerta, Contempla, Daytrana, Metadata, Ritalin, Quillichew, Quillivant, Methylin), non-stimulant drugs (i.e. Atomoxetine, Guanfacine, Clonidine, Strattera, Tenex, Intuniv, Catapres, Kapvay), and no medication. Therefore, subjects' medication conditions could be summarized into 4 groups: 0-no medication, 1-stimulant only, 2-non-stimulant medication only, 3-stimulant+non-stimulant medication. ADHD medication variable was transformed into 3 dummy variables to use in analyses. Other covariates used in the present study included age, sex, race, site, household income and parental education.

Method S3. Preprocessing of the AHBA data

We followed the AHBA preprocessing pipeline suggested by Arnatkevičiūtė et al. (11), including:

1) Probe-to-gene re-annotation: The assignment of probe sequences to genes was re-annotated according to the reference genome assembly GRCh38.p12 (released in 2017/12), using Re-annotator (12). We found that 45,461 (77.5%) were uniquely annotated to a gene, 3,743 (8.2%) of which differed from those provided by the AHBA. This newly re-annotated set of 45,461 probes correspond to 19,951 unique genes.
2) Data filtering: Based on the AHBA binary indicator, we excluded probes that did not exceed the background in at least 50% of all cortical and subcortical samples across all subjects. This intensity-based filtering resulted in 31,342 probes and 15,409 unique genes.
3) Probe selection: When multiple probes were used to measure the expression level of a same gene, we used the average, which was reported highly correlated with other methods in Arnatkevičiūtė et al.'s paper (11). In our data, we found a high correlation (mean Spearman rank correlation=0.88) between gene expression values selected by mean and that selected by max intensity (i.e. select probe with the highest expression level). Therefore, we used the mean in the following analyses.
4) We separated the samples into the cortical and the subcortical areas based on their MNI coordinates, using the Harvard-Oxford atlas (13), and excluded the samples located outside of the gray matter defined by this atlas (Figure S11, S13). As Arnatkevičiūtė et al.'s suggestion (11), we used samples in the left hemisphere where the samples were collected from all 6 donors, but the right hemisphere was sampled only from 2 donors.
5) Normalization: To control for the inter-individual differences, we conducted two within-donor normalizations. The expression data were first normalized within-sample and across-gene, and then normalized across samples. Given the repeatedly observed global transcriptional differences between the subcortical and the cortical regions, the normalizations were conducted separately for the samples from the subcortical and the cortical tissues (14, 15) (Figure S12, S14). One gene failed the normalization and therefore was deleted, resulting 15,408 genes.

Method S4. CLPM and MRI analysis considering the family relatedness based on genetic data

In addition to the family relatedness obtained from a questionnaire provided by ABCD dataset ("acspsw02"), we also used SNP data to robustly infer the family relationships. The KING package (16) was used to estimate the kinship coefficients (17, 18). An estimated kinship coefficient range >0.354, [0.177, 0.354], [0.0884, 0.177] corresponds to MZ twin, 1st-degree, and 2nd-degree. The DZ twin and full-sibling were distinguished by the ages. In our analysis, we excluded 42 participants whose genetic data were missing in the current release of the ABCD data set, and another 3 participants with inconsistent family relationships between those recorded in the questionnaire and those reconstructed from the genetic kinship (i.e. kinship was too high to be unrelated participants). The Matlab code for the multi-level block permutation is available at https://github.com/qluo2018/FamilyPermutationABCD.

For the cross-lagged panel analysis, we found 3,036 participants had complete baseline and follow-up behavioral data and qualified genetic data, resulting in 2,358 singles, 210 MZ twins, 314 DZ twins, 142 full-siblings and 12 half-siblings. We used multi-level block permutation (19) (5,000 times) to test the significance (denoted by P_{perm}). The results were consistent with that using the questionnaire (ADHD→total sleep: $P_{perm}<0.001$; total sleep→ADHD: $P_{perm}=0.008$; ADHD→dyssomnia: $P_{perm}<0.001$; dyssomnia→ADHD: $P_{perm}=0.024$; ADHD→parasomnia: $P_{perm}<0.001$; parasomnia→ADHD: $P_{perm}=0.034$).

For the MRI analysis, we found 3,470 out of 3,515 participants had qualified genetic data (i.e., 2,693 singles, 240 MZ twins, 351 DZ twins, 168 full-siblings and 18 half-siblings). Linear models with age, sex, handedness, race, puberty, BMI, site, household income, parental education, head motion and TIV as covariates of no interest were used. We conducted a multi-level block permutation-based cluster-level correction (5,000 times) for multiple comparisons. At voxel level, we used a two-sided test with a significance level of α=0.001, whereas, at cluster level, we used a permutation-based family-wise error (FWE) correction with α=0.05. Finally, we found similar significant regions associated with ADHD and dyssomnia as that using the questionnaire (Table S3, Table S5 and Figure S7).

Method S5. Meta-analyses of path coefficients in each ABCD site

To test the robustness of our findings in the ABCD cohort, we conducted cross-lagged panel models (CLPM) in each site respectively. In each model, we controlled for several stable variables (i.e. sex and race) and time-variant parameters (i.e. ADHD medication, household income, educational level of parents, BMI and puberty). Then we performed two meta-analyses of the path coefficients of both ADHD→sleep and sleep→ADHD using random-effects models by the metafor R package (20). Samples in 3 sites were too small to estimate CLPMs, so the final meta-analyses included data from 18 sites.

Result S1. RI-CLPM using the same participants at age 8 and 10 years

In QLSCD, there are different missing data in different measurement waves. In order to make sure that the main results in QLSCD is not a cohort effect, we performed random-intercepts cross-lagged panel model using the participants who had data at both ages 8 and 10 years only (n=1,263). We found similar results as using 1,601 samples. The only significant cross-lagged association was between ADHD symptoms at age 8 and sleep problems at age 10 (standardized β=0.09, 95% CI [0.01,0.18]), and this association remained significant after controlling for sex, ADHD medication and SES (n=1,213; standardized ß=0.11, 95% CI [0.02,0.19]).

Result S2. CLPMs using children with ADHD in the ABCD cohort

Based on the ABCD Parent Diagnostic Interview for DSM-5 Full (KSADS-5) Diagnostic (abcd_ksad01), we found 281 participants were diagnosed as ADHD at baseline. By conducting the same analysis as the main text (i.e. controlling for sex, race, site, ADHD medication, household income, education level of parents, BMI and puberty), we have confirmed that the cross-lagged path of ADHD→sleep was significant (sleep total score: β=0.10, 95% CI [0.01, 0.20]), while the cross-lagged path of sleep→ADHD was not significant (sleep total score: β=0.02, 95% CI [-0.05, 0.09]).

Result S3. PLS results

The selection of PLS components is based on the amount of explained variable, which is tested by permuting the response variables 5,000 times. In subcortical regions, the first component (PLS1) explained 30% of the mediation effect (y; $p<0.001$), and the second component (PLS2) explained 15% of y ($p=0.36$). PLS1 was significantly associated with y ($r=0.55$, $p<0.001$). In cortical regions, PLS1 explained 8% of y ($p<0.001$), and PLS2 explained 5% of y ($p=0.32$). PLS1 was significantly associated with y ($r=0.28$, $p<0.001$). Therefore, we only extracted PLS1 and used the weight of PLS1 to estimate the significance of genes. A Z score was calculated for each weight in a PLS component as the ratio between each weight estimation and the standard error (SE) given by 5,000 bootstraps. Therefore, the genes could be ranked by their normalized contributions to the PLS component (**Table S15A-B** list the genes ranked for the subcortical and the cortical regions, respectively).

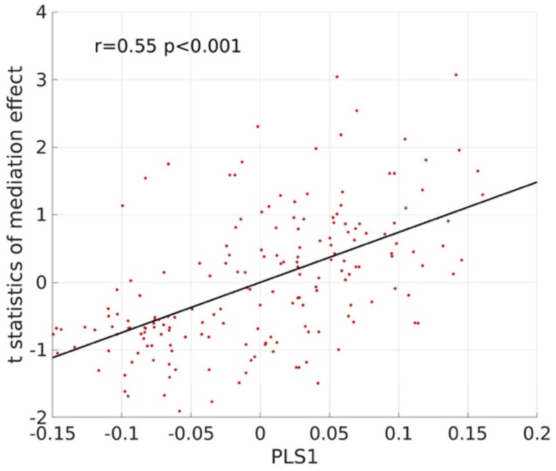

Scatterplot of PLS1 scores in subcortical regions vs. mediation effects

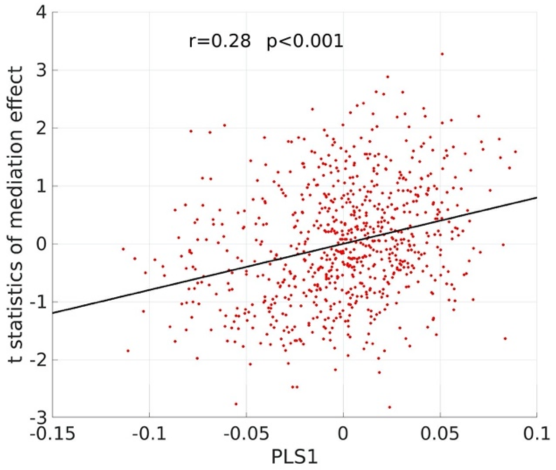

Scatterplot of PLS1 scores in cortical regions vs. mediation effects

Result S4. Leave-one-out validation of transcriptomic analysis

We used gene expression data from Allen Human Brain Atlas (AHBA; http://human.brain-map.org). This dataset includes samples from post-mortem brain of six donors (3 Caucasian, 2 African-American, 1 Hispanic) aged 24-57 years. As the limited sample size and the large variability in age, gender and ethnicity, there may be potential inter-individual differences of transcriptional patterns. Therefore, we used leave-one-donor-out approach to test the influence of individual donors on the partial least square (PLS) results. The PLS were performed six times by leaving samples from one donor out. The variance of the PLS1 weight was established for each gene by 5,000 bootstraps, and the ratio between the weight of each gene to its bootstrap standard error (SE) was used to calculate the Z scores. PLS regressions using subcortical and cortical samples were conducted separately.

For subcortical regions, we found the variance explained by PLS1 was significant at each time (all>28%). And the correlations of PLS1 Z scores were high (all r>0.95). The consistency of PLS results confirms that PLS results in subcortical regions reported in the present study were not driven by a single donor.

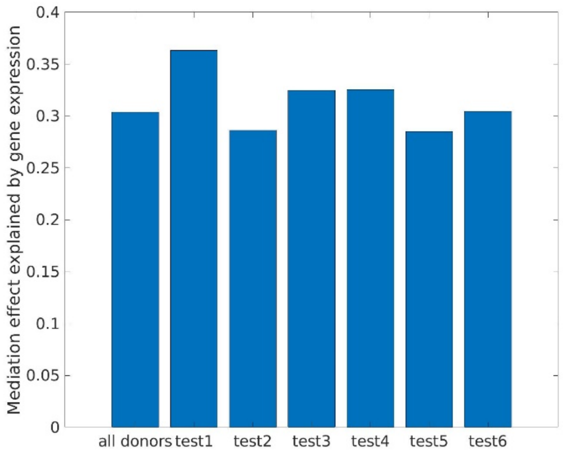

The variance explained by PLS1 using all six donors and 5 donors in subcortical regions

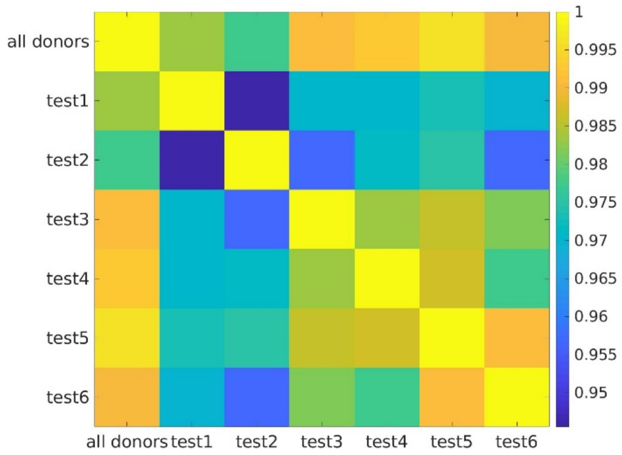

Correlations of PLS1 Z scores using all six donors and 5 donors in subcortical regions

For cortical regions, we also found the variance explained by PLS1 was significant at each time (all>=8%). And the correlations of PLS1 Z scores were high (all r>0.8). The consistency of PLS results confirms that PLS results in cortical regions reported in the present study were not driven by a single donor.

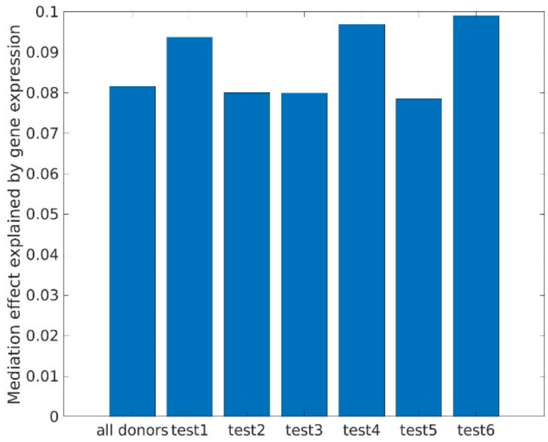

The variance explained by PLS1 using all six donors and 5 donors in cortical regions

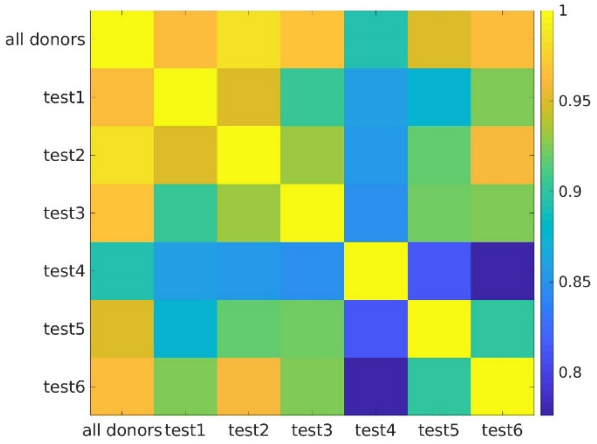

Correlations of PLS1 Z scores using all six donors and 5 donors in cortical regions

Result S5. Validation of transcriptomic analysis using 6mm ROIs.

The dependent variable used in PLS regression was calculated by the averaged mediation effect t value of a spherical region of interests (ROI; r=4mm) centered by the MNI coordinates of each gene expression sampling site (21). In order to test whether the findings depended on the size of ROI, we extracted t values based on 6mm ROIs and performed PLS using the same pipeline here.

In subcortical regions, there were 109 available samples (23, 21, 20, 18, 9, 18, respectively). The correlation of PLS1 Z scores between 4mm ROI and 6mm ROI was high (r=0.94, p<0.001), suggesting that the PLS results in subcortical regions were not driven by the definition of ROI.

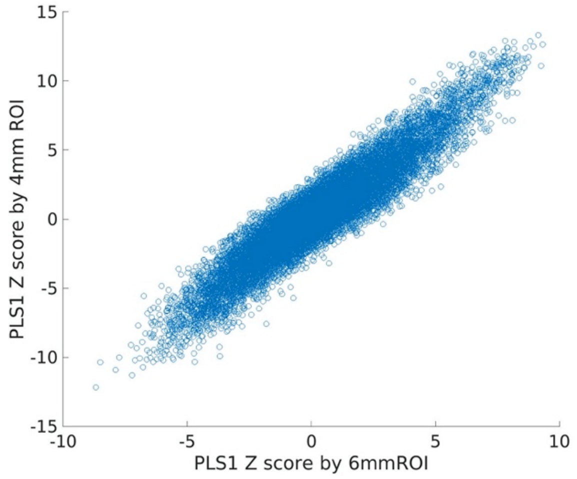

Correlations of PLS1 Z scores by 4mm ROI and by 6mm ROI in subcortical regions

In cortical regions, there were 635 available samples (115, 93, 91, 114, 114, 108, respectively). The correlation of PLS1 Z scores between 4mm ROI and 6mm ROI was high (r=0.93, p<0.001), suggesting that the PLS results in cortical regions were not driven by the definition of ROI.

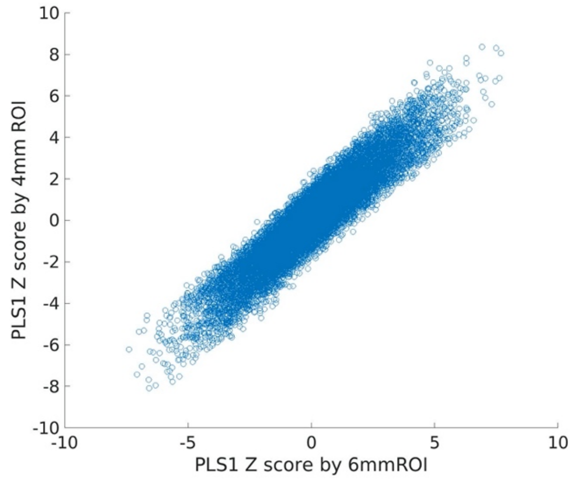

Correlations of PLS1 Z scores by 4mm ROI and by 6mm ROI in cortical regions

Result S6. Validation of transcriptomic analysis in cortical regions using a refined brain atlas.

In order to examine whether the PLS results were biased by the definition of ROI, we conducted another PLS analysis using a refined cortical brain atlas (22, 23), and compared with the PLS results using 4-mm ROIs. There are 152 cortical regions in the left hemisphere. Tissue samples were pooled based on the MNI coordinates released by the AHBA. Regional expression levels for each gene were averaged by corresponding samples. We found a significant association between PLS1 Z scores calculated by 4-mm cortical ROIs and PLS1 Z scores calculated by the cortical atlas (152 cortical regions) (r=0.59, p<0.001).

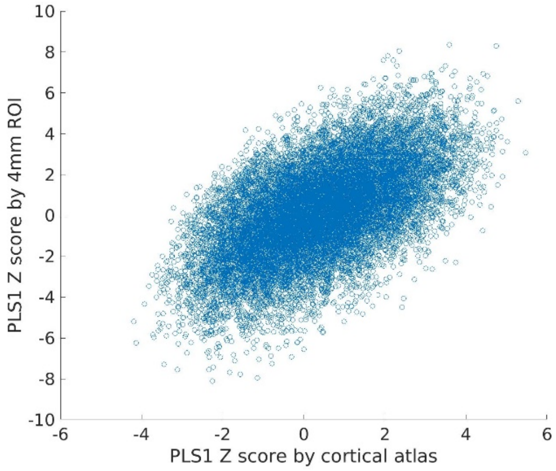

Correlations of PLS1 Z scores by a refined cortical atlas and by 4mm ROI in cortical regions

Table S1. Results of FDR correction of RI-CLPM in QLSCD.

From	To	p.fdr	Zstats
Sleep 12y	Sleep 13y	0.9879	0.02
ADHD 12y	Sleep 13y	0.8490	0.71
Sleep 10y	Sleep 12y	<0.0001	19.50
ADHD 10y	Sleep 12y	0.9879	-0.02
Sleep 8y	Sleep 10y	<0.0001	12.57
ADHD 8y	Sleep 10y	0.0493	2.36
Sleep 7y	Sleep 8y	0.9097	-0.41
ADHD 7y	Sleep 8y	0.5308	1.19
ADHD 12y	ADHD 13y	0.0118	2.97
Sleep 12y	ADHD 13y	0.8018	0.84
ADHD 10y	ADHD 12y	0.9879	-0.15
Sleep 10y	ADHD 12y	0.9439	0.30
ADHD 8y	ADHD 10y	0.0187	2.76
Sleep 8y	ADHD 10y	0.9097	-0.44
ADHD 7y	ADHD 8y	<0.0001	7.36
Sleep 7y	ADHD 8y	0.8754	-0.60

Table S2. Significant GMVs associated with ADHD symptoms corrected for the family relatedness defined by the questionnaire.

	Cluster size (k)	MNI (at peak)			T-value	pFWE[a]
		x	y	z		
Middle frontal gyrus, superior frontal gyrus, inferior frontal gyrus, temporal lobe, insula, anterior cingulate, caudate, thalamus, parahippocampus, hippocampus, putamen, occipital lobe, amygdala	64,401	38	41	17	-5.87	<0.001
Middle frontal gyrus, inferior frontal gyrus, insula	1,465	42	2	20	-4.03	0.031

N=3,515. Covariates: sex, age, puberty, BMI, handedness, maternal education, family income, race, TIV, head motion score and site.

a: multiple comparison correction with an uncorrected, two-tailed $p<0.001$ at voxel level and a cluster-level family-wise error (FWE) $p<0.05$; the pFWE was obtained by comparing the real cluster size with the null distribution of maximum cluster size which was estimated by 5,000 multi-level block permutations considering family relatedness (i.e., single, sibling, twin and triple; from questionnaire).

Table S3. Significant GMVs associated with ADHD symptoms corrected for the family relatedness reconstructed from the genetic kinship.

	Cluster size (k)	MNI (at peak)			T-value	pFWE[a]
		x	y	z		
Middle frontal gyrus, superior frontal gyrus, inferior frontal gyrus, temporal lobe, insula, anterior cingulate, caudate, thalamus, parahippocampus, hippocampus, putamen, amygdala	49,692	38	41	17	-5.82	<0.001
Occipital lobe, lingual gyrus, temporal lobe	10,169	38	-86	12	-5.02	0.003

N=3,470. Covariates: sex, age, puberty, BMI, handedness, maternal education, family income, race, TIV, head motion score and site.

a: multiple comparison correction with an uncorrected, two-tailed p<0.001 at voxel level and a cluster-level family-wise error (FWE) p<0.05; the pFWE was obtained by comparing the real cluster size with the null distribution of maximum cluster size which was estimated by 5,000 multi-level block permutations considering family relatedness (i.e., single, MZ, DZ, full-sibling and half-sibling; from SNP data).

Table S4. Significant GMVs associated with dyssomnia corrected for the family relatedness defined by the questionnaire.

	Cluster size (k)	MNI (at peak)			T-value	pFWE[a]
		x	y	z		
Insula, caudate, putamen, inferior frontal gyrus	2,948	-8	8	3	-4.51	0.014
Middle frontal gyrus, insula	2,932	36	41	17	-4.18	0.014
Fusiform, parahippocampus, amygdala, right hippocampus	2,144	41	-29	-29	-4.77	0.021

N=3,515. Covariates: sex, age, puberty, BMI, handedness, maternal education, family income, race, TIV, head motion score and site.

a: multiple comparison correction with an uncorrected, two-tailed p<0.001 at voxel level and a cluster-level family-wise error (FWE) p<0.025; the pFWE was obtained by comparing the real cluster size with the null distribution of maximum cluster size which was estimated by 5,000 multi-level block permutations considering family relatedness (i.e., single, sibling, twin and triple; from questionnaire).

Table S5. Significant GMVs associated with dyssomnia corrected for the family relatedness reconstructed from the genetic kinship.

	Cluster size (k)	MNI (at peak)			T-value	pFWE [a]
		x	y	z		
Middle frontal gyrus, infior frontal gyrus, insula	4,764	35	42	15	-4.25	0.008
Insula, caudate, inferior frontal gyrus, putamen	3,198	-8	8	3	-4.56	0.015
Temporal lobe, parahippocampus, amygdala, right hippocampus	2,396	41	-29	-29	-4.84	0.022

N=3,470. Covariates: sex, age, puberty, BMI, handedness, maternal education, family income, race, TIV, head motion score and site.

a: multiple comparison correction with an uncorrected, two-tailed p<0.001 at voxel level and a cluster-level family-wise error (FWE) p<0.025; the pFWE was obtained by comparing the real cluster size with the null distribution of maximum cluster size which was estimated by 5,000 multi-level block permutations considering family relatedness (i.e., single, MZ, DZ, full-sibling and half-sibling; from SNP data).

Table S6. Significant biological processes and KEGG pathways of the top and bottom 1% genes in subcortical and cortical regions.

Significant enrichments of the top 1% negatively correlated genes in the subcortical regions				
**Category**	**Description**	**Gene Count**	**Raw p-value**	**FDR q-value**
GOTERM_BP_DIRECT	Chemical synaptic transmission	13	2.0E-7	1.3E-4
GOTERM_BP_DIRECT	Innervation	5	7.7E-6	2.6E-3
KEGG_PATHWAY	Oxytocin signaling pathway	10	7.1E-7	9.7E-5
KEGG_PATHWAY	Nicotine addiction	6	7.3E-6	2.5E-4
KEGG_PATHWAY	Long-term potentiation	7	5.5E-6	2.5E-4
KEGG_PATHWAY	Retrograde endocannabinoid signaling	8	5.4E-6	3.7E-4
KEGG_PATHWAY	Amphetamine addiction	6	4.8E-4	1.1E-2
KEGG_PATHWAY	Circadian entrainment	6	4.8E-4	1.1E-2
KEGG_PATHWAY	Glioma	5	1.0E-3	2.0E-2
KEGG_PATHWAY	Adherens junction	5	1.4E-3	2.4E-2
KEGG_PATHWAY	ErbB signaling pathway	5	3.0E-3	2.7E-2
KEGG_PATHWAY	Neuroactive ligand-receptor interaction	8	2.8E-3	2.7E-2
KEGG_PATHWAY	Dopaminergic synapse	6	1.9E-3	2.8E-2
KEGG_PATHWAY	Proteoglycans in cancer	7	2.5E-3	2.8E-2
KEGG_PATHWAY	cAMP signaling pathway	7	2.4W-3	2.9E-2
KEGG_PATHWAY	GABAergic synapse	5	2.8E-3	2.9E-2
KEGG_PATHWAY	Morphine addiction	5	3.6E-3	3.0E-2
KEGG_PATHWAY	Aldosterone synthesis and secretion	5	2.3E-3	3.2E-2
KEGG_PATHWAY	cGMP-PKG signaling pathway	6	4.6E-3	3.7E-2
KEGG_PATHWAY	Melanogenesis	5	5.0E-3	3.8E-2
KEGG_PATHWAY	Long-term depression	4	8.3E-3	5.0E-2
Significant enrichments of the top 1% positively correlated genes in the subcortical regions				
**Category**	**Description**	**Gene Count**	**Raw p-value**	**FDR q-value**
GOTERM_BP_DIRECT	Central nervous system myelination	4	1.9E-5	1.6E-2
Significant enrichments of the top 1% negatively correlated genes in the cortical regions				
**Category**	**Description**	**Gene Count**	**Raw p-value**	**FDR q-value**
GOTERM_BP_DIRECT	Transcription, DNA-templated	46	1.2E-11	7.5E-9
GOTERM_BP_DIRECT	Regulation of transcription, DNA-templated	38	2.4E-10	7.4E-8
Significant enrichments of the top 1% positively correlated genes in the cortical regions				
**Category**	**Description**	**Gene Count**	**Raw p-value**	**FDR q-value**
KEGG_PATHWAY	Biosynthesis of antibiotics	11	6.4E-6	9.2E-4
KEGG_PATHWAY	Valine, leucine and isoleucine degradation	6	3.5E-5	2.5E-3
KEGG_PATHWAY	Carbon metabolism	7	2.9E-4	1.4E-2

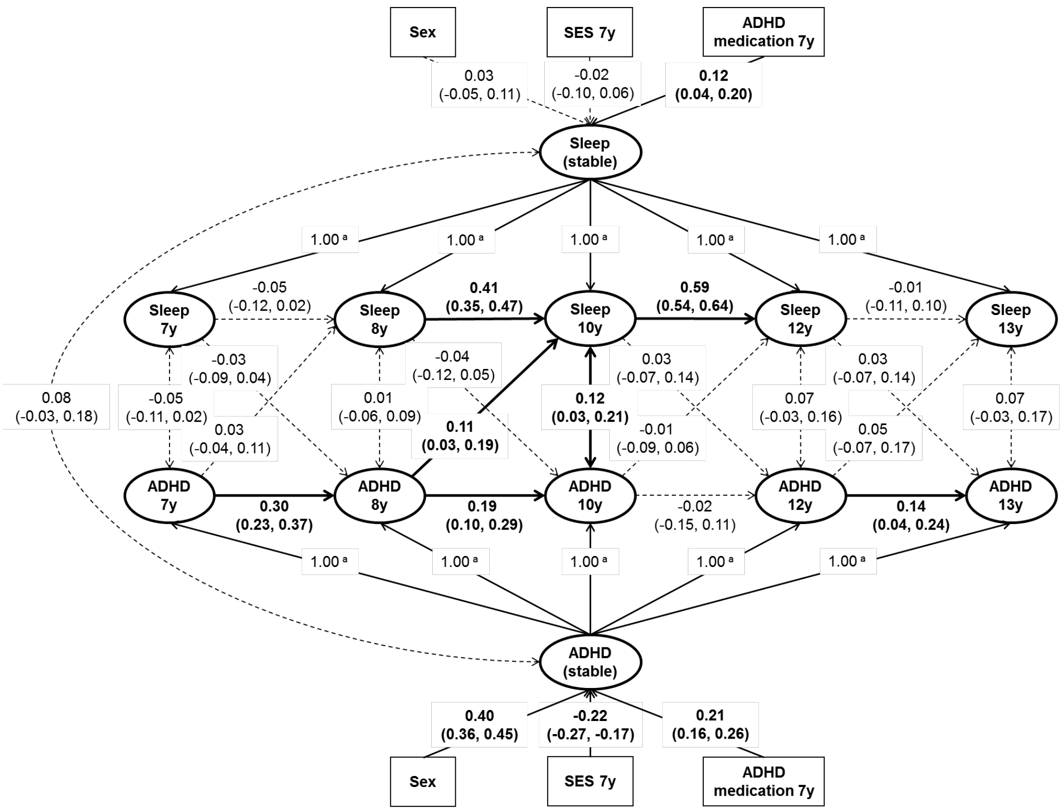

Figure S1. RI-CLPM of ADHD total score and sleep disturbance from ages 7 to 13 years in QLSCD with covariates.

N=1,467. Covariates included sex, SES and ADHD medication at age 7 years. Standardized estimates (95% CIs) are presented. Solid lines represent statistically significant ($p<0.05$), whereas dashed lines present unsignificant. Model fit: RMSEA=0.04; CFI=0.97; TLI=0.96; SRMR=0.03. In this model, we found the ADHD medication at age 7 was positively associated with both the overall sleep disturbance (β=0.12, 95%CI [0.04, 0.20]) and ADHD symptoms (β=0.21, 95%CI [0.16, 0.26]), sex was significantly associated with the overall ADHD (β=0.40, 95%CI [0.36, 0.45]) but not sleep disturbance (β=0.03, 95%CI [-0.05, 0.11]).

[a] Pathways constrained to 1.00 to isolate between-person factor.

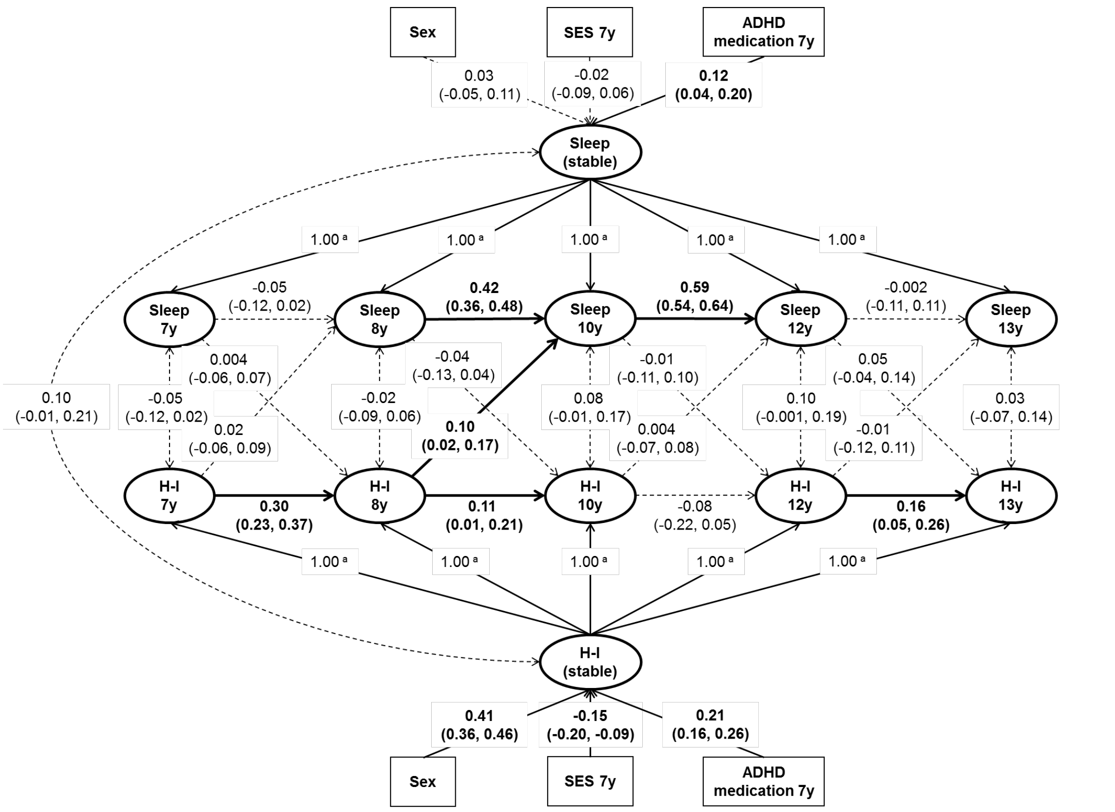

Figure S2. RI-CLPM of hyperactivity-impulsivity symptom and sleep disturbance from ages 7 to 13 years in QLSCD with covariates.

N=1,467. Covariates included sex, SES and ADHD medication at age 7 years. Standardized estimates (95% CIs) are presented. Solid lines represent statistically significant ($p<0.05$), whereas dashed lines present unsignificant. Model fit: RMSEA=0.04; CFI=0.97; TLI=0.95; SRMR=0.03.
[a] Pathways constrained to 1.00 to isolate between-person factor.

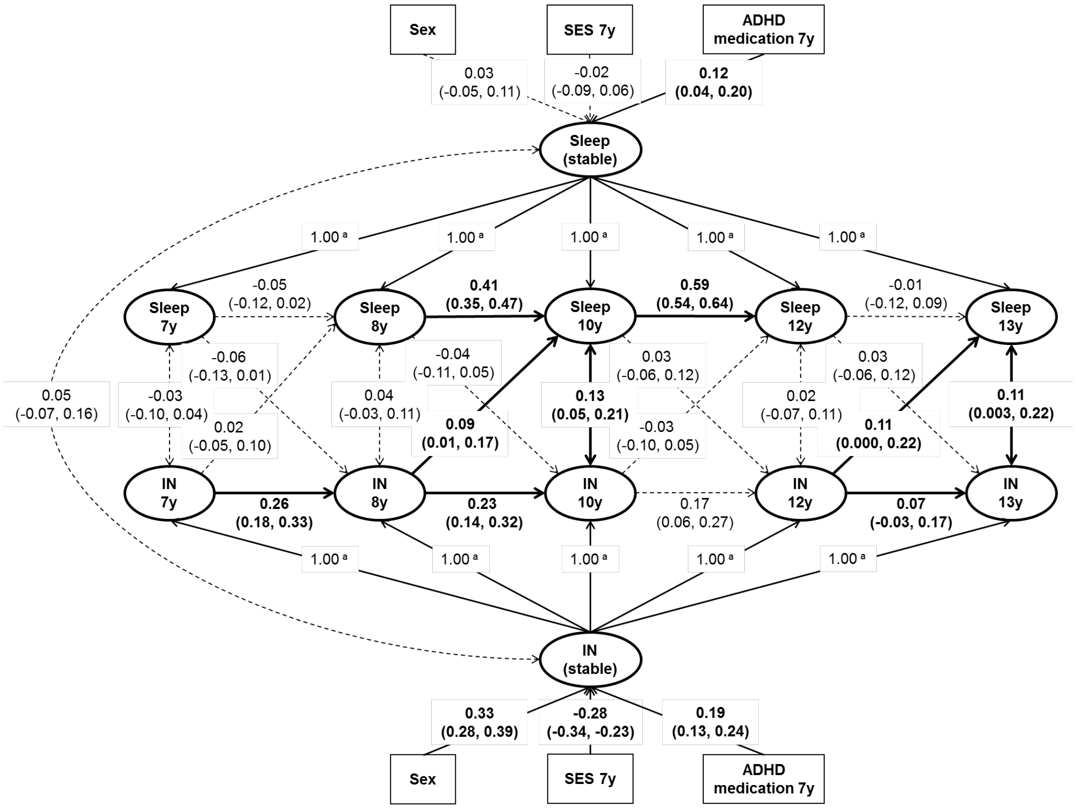

Figure S3. RI-CLPM of inattention symptom and sleep disturbance from ages 7 to 13 years in QLSCD with covariates.

N=1,467. Covariates included sex, SES and ADHD medication at age 7 years. Standardized estimates (95% CIs) are presented. Solid lines represent statistically significant ($p<0.05$), whereas dashed lines present unsignificant. Model fit: RMSEA=0.03; CFI=0.98; TLI=0.96; SRMR=0.03.
[a] Pathways constrained to 1.00 to isolate between-person factor.

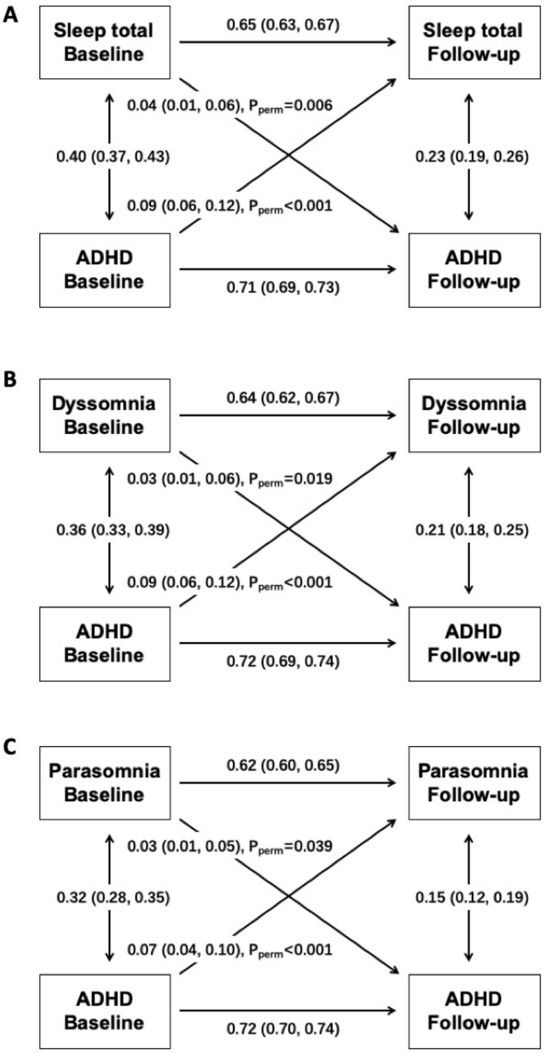

Figure S4. CLPMs of ADHD symptoms and sleep disturbance from baseline to follow-up in the ABCD study.

N=3,076. Covariates included sex, BMI, puberty, parental education, household income, ADHD medication, site and race. Standardized estimates (95% CIs) are presented. Solid lines represent statistically significant ($p<0.05$), whereas dashed lines present unsignificant. P_{perm} was estimated by 5,000 times multi-level block permutation considering family relatedness (i.e., single, sibling, twin and triple; from questionnaire). For the model of ADHD and total sleep, P_{perm} was corrected by combining paths of ADHD→total sleep and total sleep→ADHD. For the models of ADHD and sleep dimension scores, P_{perm} was corrected by combining paths of ADHD→dyssomnia, dyssomnia→ADHD, ADHD→parasomnia and parasomnia→ADHD. A. CLPM of ADHD and sleep total score. Model fit: RMSEA=0.04, CFI=0.98, TLI=0.91, SRMR=0.005. The model accounted for 61% of variance in ADHD and 50.3%% of variance in sleep total score at the follow-up. B. CLPM of ADHD and dyssomnia. Model fit: RMSEA=0.04, CFI=0.98, TLI=0.90, SRMR=0.005. The model accounted for 61% of variance in ADHD and 48.4% of variance in

dyssomnia at the follow-up. C. CLPM of ADHD and Parasomnia. Model fit: RMSEA=0.04, CFI=0.98, TLI=0.90, SRMR=0.004. The model accounted for 60.9% of variance in ADHD and 44.6%% of variance in parasomnia at the follow-up.

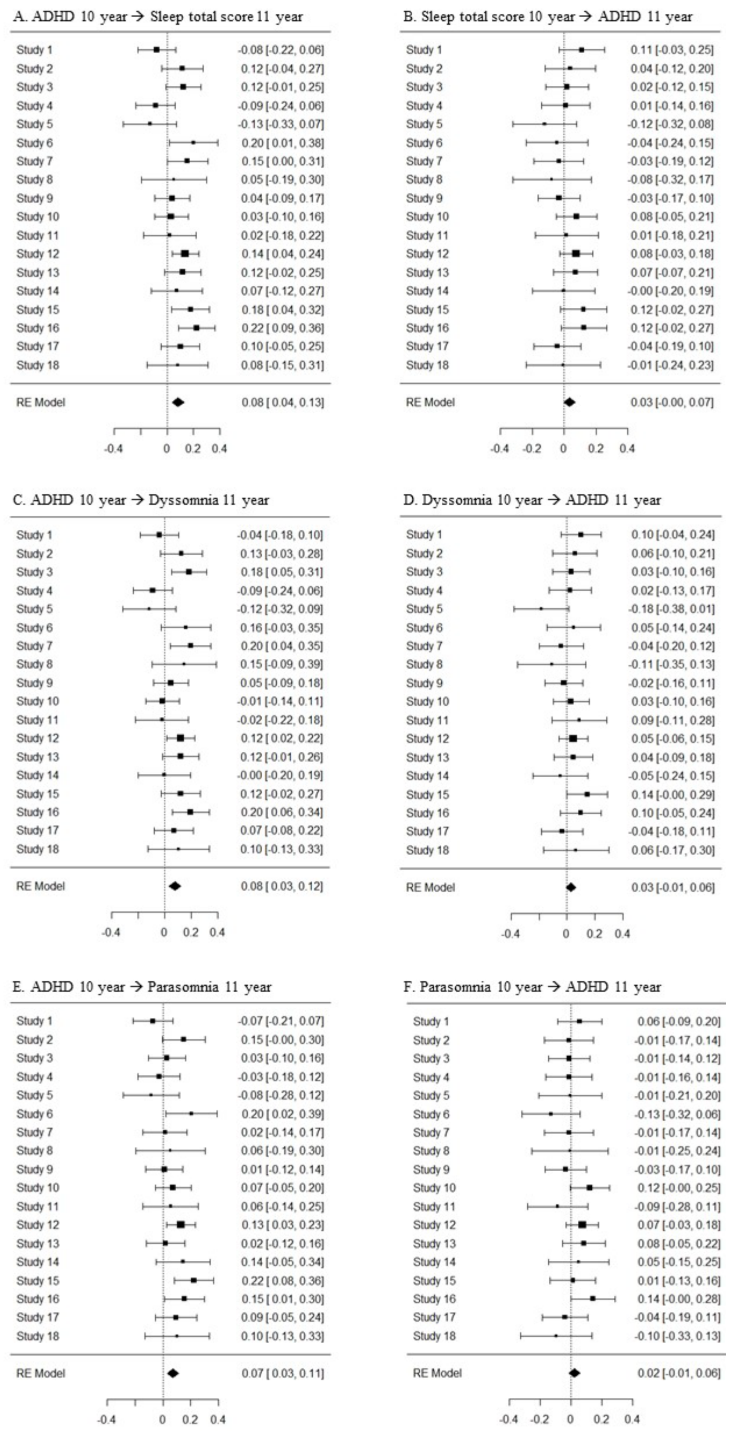

Figure S5. Forest plots of meta-analyses of path coefficients across 18 data collection sites in the ABCD cohort.

Results were estimated by random-effects model. A. Path ADHD at age 10→sleep total score at age 11. B. Path sleep total score at age 10→ADHD at age 11. C. Path ADHD at age 10→dyssomnia at

age 11. D. Path dyssomnia at age 10→ADHD at age 11. E. Path ADHD at age 10→parasomnia at age 11. F. Path parasomnia at age 10→ADHD at age 11.

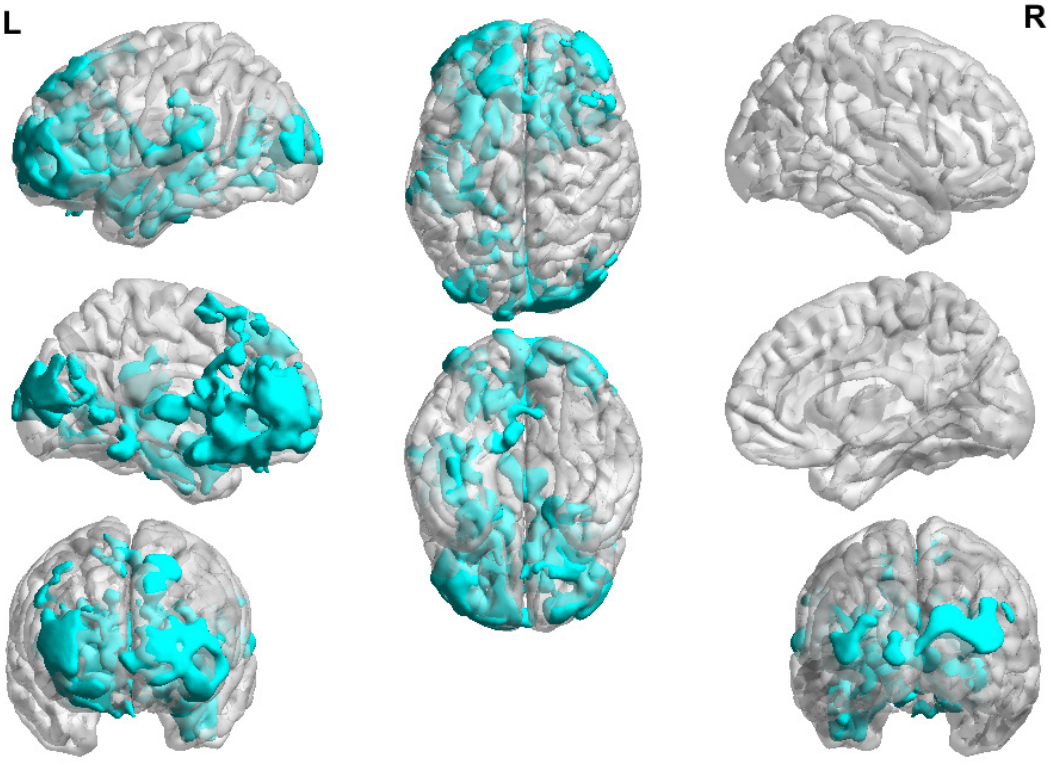

Figure S6. 3-D view of significant GMVs associated with ADHD symptoms corrected for the family relatedness defined by the questionnaire.

N=3,515. Two large clusters were found negatively associated with ADHD symptoms visualized with the BrainNet Viewer (24).

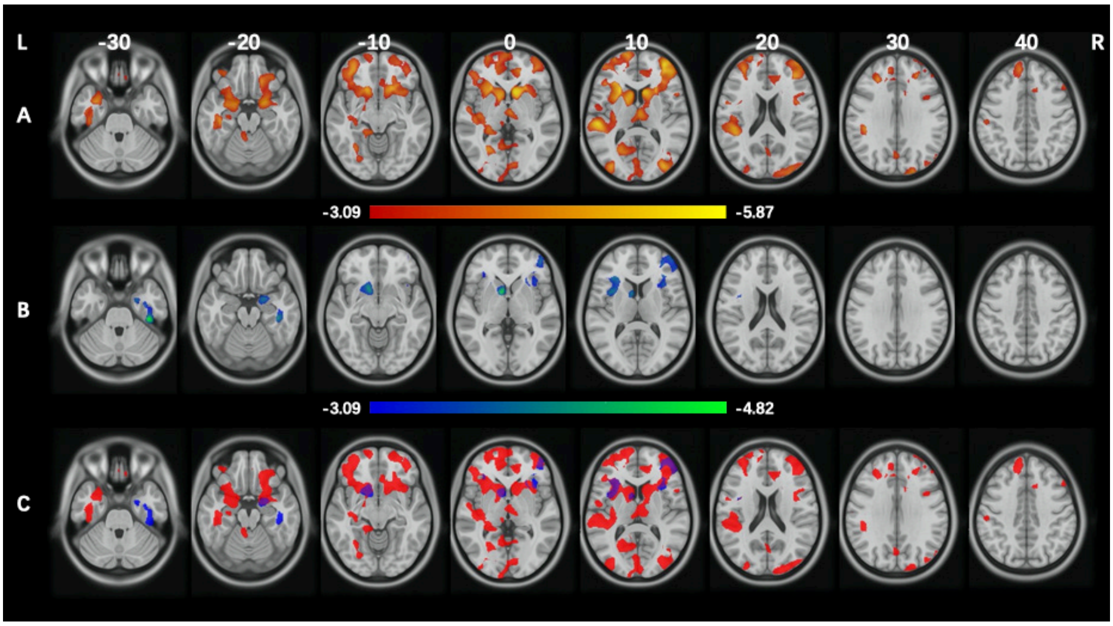

Figure S7. Significant brain clusters associated with ADHD symptoms and dyssomnia corrected for the family relatedness reconstructed from the genetic kinship

N=3,470. Significance was tested by multi-level block permutation-based cluster-level correction (5,000 times) (family relationships estimated by SNP data). At voxel level, we used a two-sided test with a significance level of α=0.001, whereas, at cluster level, we used a permutation-based family-wise error (FWE) correction with α=0.05. A. Brain regions significantly associated with ADHD symptoms. Multiple comparison correction includes voxel-level p<0.001 and cluster-level pFWE<0.05. The color bar represents t-value. B. Brain regions significantly associated with dyssomnia. Multiple comparison correction includes voxel-level p<0.001 and cluster-level pFWE<0.025. The color bar represents t-value. C. Brain regions significantly associated with ADHD or dyssomnia. Red areas are associated with ADHD, blue areas are associated with dyssomnia, and purple areas are the overlapping regions. Age, sex, handedness, race, puberty, BMI, site, household income, parental education, head motion and TIV were controlled for in all analyses.

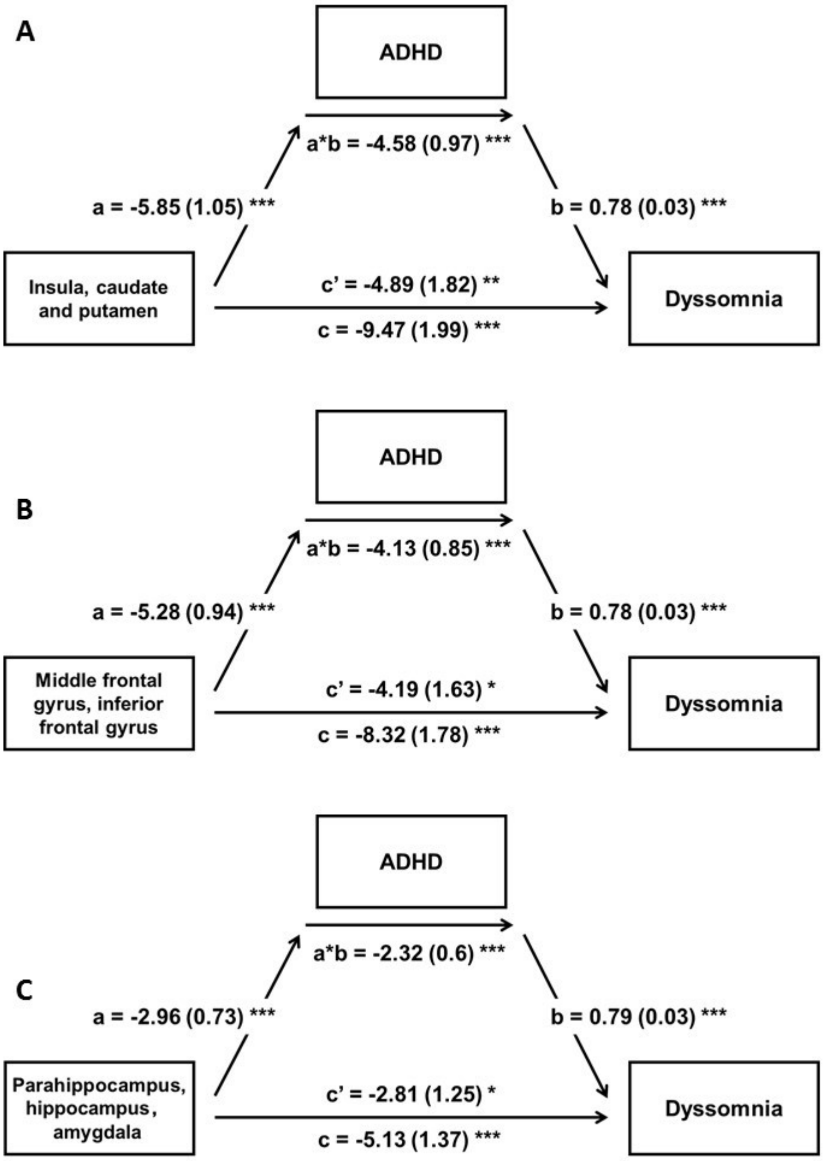

Figure S8. Mediation models of average GMV in 3 overlapping clusters separately.

A. Mediation analysis using average GMV of the striatum cluster (2,762 voxels, including caudate, putamen, insula) as independent variable, the indirect to total effect is 48.4% [30.6%,79.2%]. B. Mediation analysis using average GMV of the frontal cluster (2,296 voxels, including middle frontal gyrus, inferior frontal gyrus) as independent variable, the indirect to total effect is 49.7% [32.1%,82.1%]. C. Mediation analysis using average GMV of the hippocampus cluster (419 voxels, including hippocampus, parahippocampus and amygdala) as independent variable, the indirect to total effect is 45.2% [24.3%,91.1%].
$*p<0.05$, $**p<0.01$, $***p<0.001$.

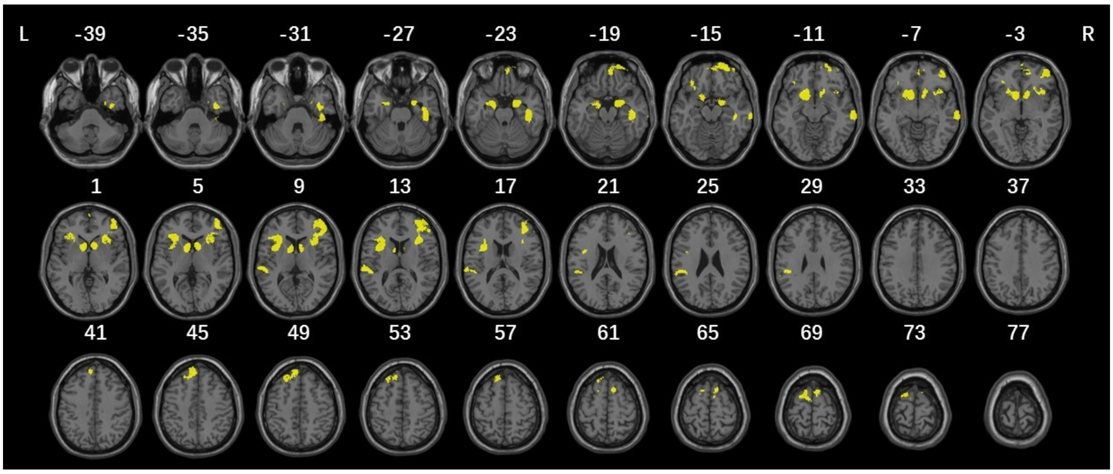

Figure S9. Results of the exploratory whole-brain voxel-wise mediation analysis.

A whole-brain and voxel-wise mediation analysis (brain→ADHD→dyssomnia) was conducted, and its significance was given by 3,000 bootstraps at each voxel and corrected by false discovery rate (FDR) correction among all voxels and a significant brain-sleep association ($p<0.005$, two-tailed, uncorrected). The yellow regions highlighted in the figure are the significant areas with more than 217 voxels, locating at frontal lobe, insula, striatum, amygdala, hippocampus, and temporal lobe. All significant voxels had a negative coefficient of a*b (mediation effect: 46.0%, 95% CI [30.9%, 69.2%]).

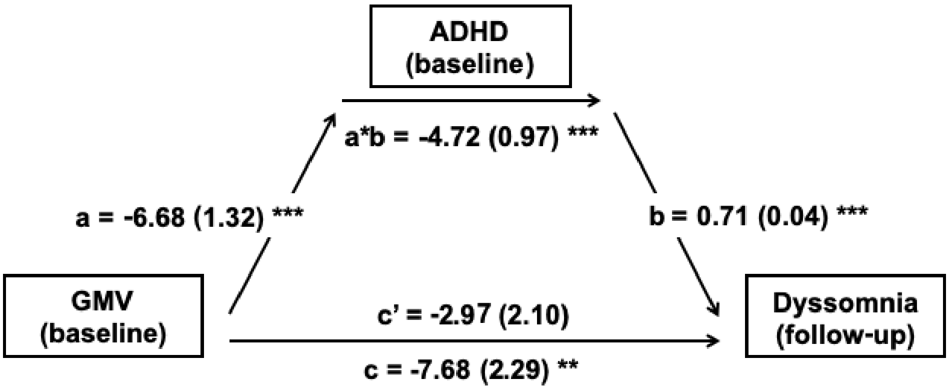

Figure S10. Associations of the baseline average overlapping gray matter volume, baseline ADHD symptoms and follow-up dyssomnia.

N=3,076. Mediation model using the baseline average overlapping GMV as the predictor, baseline ADHD as the mediator and follow-up dyssomnia as the dependent variable. Using age, sex, handedness, race, puberty, BMI, site, household income, parental education, head motion and TIV as covariates of no interest. Path a measures the association between the predictor and the mediator; path b represents the effect of the mediator on the dependent variable while controlling for the predictor; path c measures the total relationship between the predictor and the dependent variable; path c' measures the direct effect; the mediation effect is the product of path a and path b (a*b).
*p<0.05, **p<0.01, ***p<0.001.

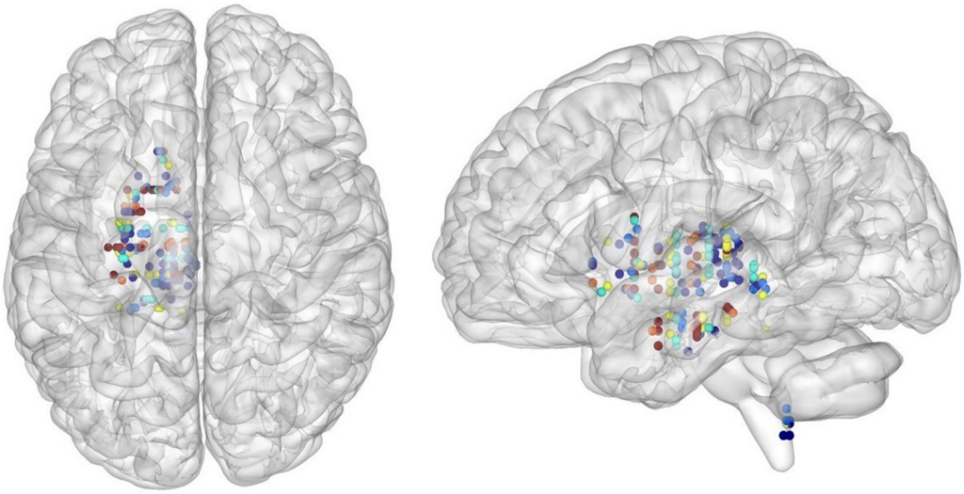

Figure S11. Subcortical tissue samples in AHBA used in partial least square regression.

As there are only two donors have samples in right hemisphere, we only included samples in left hemisphere in analysis. In total, there are 182 available samples from 6 donors (43, 36, 30, 32, 13, 28, respectively). Tissue samples from 6 donors are represented by different colors.

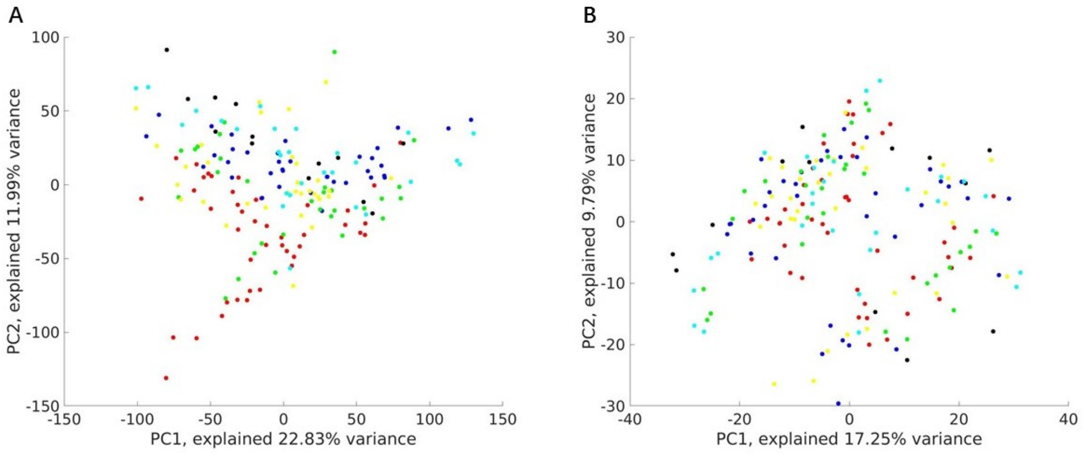

Figure S12. Subcortical gene expression data in principal component (PC) space.

Samples from six donors are represented by different colors. A. Non-normalized gene expression data in PC space. B. Scaled robust sigmoid (SRS) transformed gene expression data in PC space. We can find SRS normalization can remove donor-specific variability in gene expression.

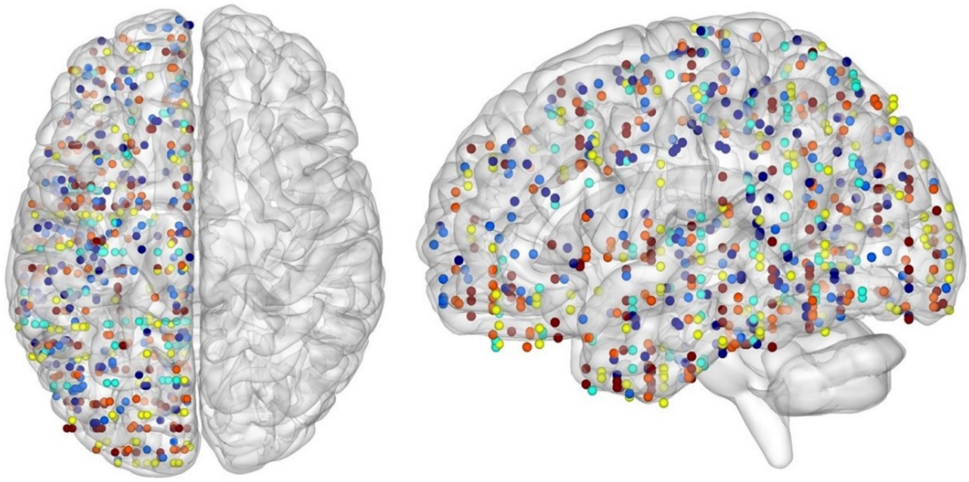

Figure S13. Cortical tissue samples in AHBA used in partial least square regression.

As there are only two donors have samples in right hemisphere, we only included samples in left hemisphere in analysis. In total, there are 784 available samples from 6 donors (135, 109, 111, 151, 136, 142, respectively). Tissue samples from 6 donors are represented by different colors.

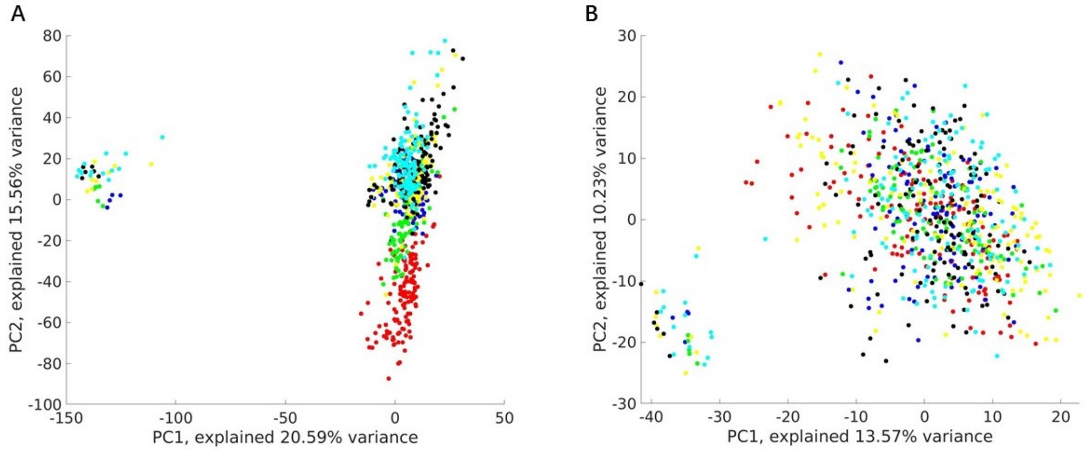

Figure S14. Cortical gene expression data in principal component (PC) space.

Samples from six donors are represented by different colors. A. Non-normalized gene expression data in PC space. B. Scaled robust sigmoid (SRS) transformed gene expression data in PC space. We can find SRS normalization can remove donor-specific variability in gene expression.

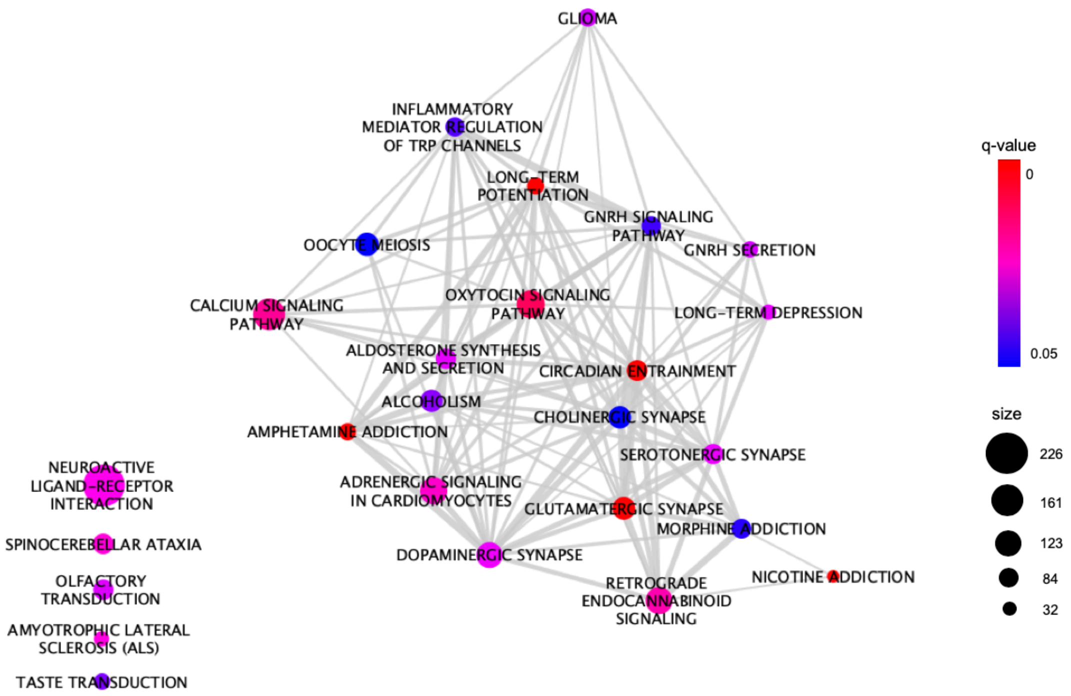

Figure S15. Enrichment network of KEGG pathways of S- gene set in subcortical regions.

Each pathway is presented by a node: node size is proportional to the number of genes in a given pathway, and color illustrates the q-value after combined correction. Edges represent gene overlapping as calculated by Jaccard coefficient (JC; intersection/union). Edges with JC equal or greater than 0.15 were presented by solid lines. The thickness of edges shows the level of overlap between two pathways. Figure was generated by cytoscape (25) for the significant KEGG enrichments of PLS1- gene set in subcortical regions (i.e. 26 pathways).

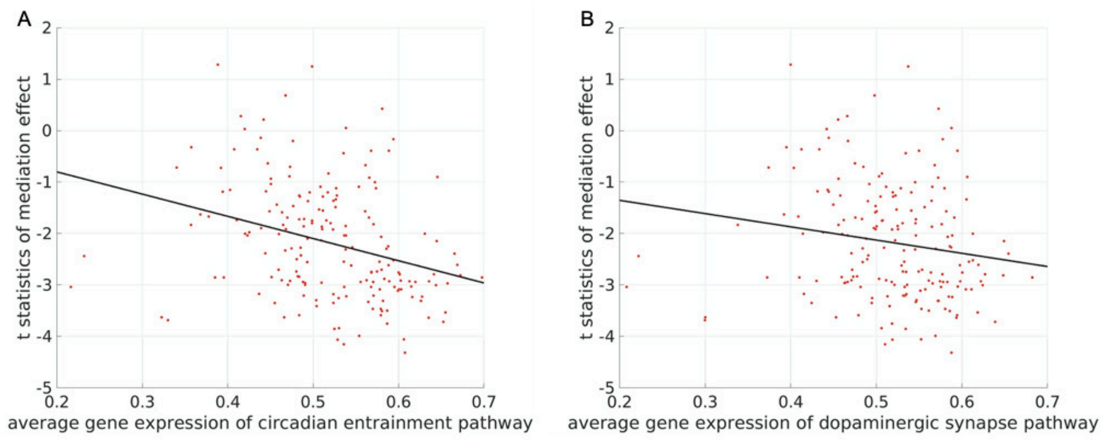

Figure S16. Scatterplots of gene expression vs. t statistics of mediation effect.

A. Scatterplot of gene expression of 88 genes in circadian entrainment pathway vs. t statistics of mediation effect. B. Scatterplot of gene expression of 123 genes in dopaminergic synapse pathway vs. t statistics of mediation effect.

Supplemental References

1. Jetté M, Des Groseilliers L (2000): Description of the Statistical Methodology of ÉLDEQ 1998-2002 (5-Month-Old Infants). *Survey Description and Methodology" in Longitudinal Study of Child Development in Québec (ÉLDEQ 1998-2002).* Québec: Institut de la statistique du Québec, pp 33-36.

2. Tremblay RE, Loeber R, Gagnon C, Charlebois P, Larivée S, LeBlanc M (1991): Disruptive boys with stable and unstable high fighting behavior patterns during junior elementary school. *J Abnorm Child Psychol* 19:285–300.

3. Battaglia M, Garon-Carrier G, Côté SM, Dionne G, Touchette E, Vitaro F, et al. (2017): Early childhood trajectories of separation anxiety: Bearing on mental health, academic achievement, and physical health from mid - childhood to preadolescence. *Depress Anxiety* 34:918-927.

4. Galera C, Cote SM, Bouvard MP, Pingault JB, Melchior M, Michel G, et al. (2011): Early risk factors for hyperactivity-impulsivity and inattention trajectories from age 17 months to 8 years. *Archives of general psychiatry* 68:1267-1275.

5. Willms J, Shields M (1996): A measure of socioeconomic status for the National Longitudinal Study of Children. *Report prepared for Statistics Canada*.

6. Achenbach TM, Ruffle TM (2000): The Child Behavior Checklist and related forms for assessing behavioral/emotional problems and competencies. *Pediatr Rev* 21:265-271.

7. Aebi M, Winkler Metzke C, Steinhausen H-C (2010): Accuracy of the DSM-oriented attention problem scale of the child behavior checklist in diagnosing attention-deficit hyperactivity disorder. *Journal of Attention Disorders* 13:454-463.

8. Bruni O, Ottaviano S, Guidetti V, Romoli M, Innocenzi M, Cortesi F, et al. (1996): The Sleep Disturbance Scale for Children (SDSC) Construct ion and validation of an instrument to evaluate sleep disturbances in childhood and adolescence. *J Sleep Res* 5:251-261.

9. Carskadon MA, Acebo C (1993): A self-administered rating scale for pubertal development. *J Adolesc Health* 14:190-195.

10. Veale JF (2014): Edinburgh handedness inventory–short form: a revised version based on confirmatory factor analysis. *Laterality: Asymmetries of Body, Brain and Cognition* 19:164-177.

11. Arnatkevic Iute A, Fulcher BD, Fornito A (2019): A practical guide to linking brain-wide gene expression and neuroimaging data. *Neuroimage* 189:353-367.

12. Arloth J, Bader DM, Roh S, Altmann A (2015): Re-Annotator: Annotation Pipeline for Microarray Probe Sequences. *PLoS One* 10.

13. Desikan RS, Ségonne F, Fischl B, Quinn BT, Dickerson BC, Blacker D, et al. (2006): An automated labeling system for subdividing the human cerebral cortex on MRI scans into gyral based regions of interest. *Neuroimage* 31:968-980.

14. Hawrylycz MJ, Lein ES, Guillozet-Bongaarts AL, Shen EH, Ng L, Miller JA, et al. (2012): An anatomically comprehensive atlas of the adult human brain transcriptome. *Nature* 489:391-399.

15. Anderson KM, Krienen FM, Choi EY, Reinen JM, Yeo BTT, Holmes AJ (2018): Gene expression links functional networks across cortex and striatum. *Nat Commun* 9:1428.

16. Manichaikul A, Mychaleckyj JC, Rich SS, Daly K, Sale M, Chen WM (2010): Robust relationship inference in genome-wide association studies. *Bioinformatics* 26:2867-2873.

17. Bycroft C, Freeman C, Petkova D, Band G, Elliott LT, Sharp K, et al. (2018): The UK Biobank resource with deep phenotyping and genomic data. *Nature* 562:203-209.

18. Kochunov P, Donohue B, Mitchell BD, Ganjgahi H, Adhikari B, Ryan M, et al. (2019): Genomic kinship construction to enhance genetic analyses in the human connectome project data. *Hum Brain Mapp* 40:1677-1688.

19. Winkler AM, Webster MA, Vidaurre D, Nichols TE, Smith SM (2015): Multi-level block permutation. *Neuroimage* 123:253-268.

20. Viechtbauer W (2010): Conducting meta-analyses in R with the metafor package. *J Stat Softw* 36.

21. Albaugh MD, Orr C, Chaarani B, Althoff RR, Allgaier N, D'Alberto N, et al. (2017): Inattention and reaction time variability are linked to ventromedial prefrontal volume in adolescents. *Biol Psychiatry* 82:660-668.

22. Romero-Garcia R, Atienza M, Clemmensen LH, Cantero JL (2012): Effects of network resolution on topological properties of human neocortex. *Neuroimage* 59:3522-3532.

23. Morgan SE, Seidlitz J, Whitaker KJ, Romero-Garcia R, Clifton NE, Scarpazza C, et al. (2019): Cortical patterning of abnormal morphometric similarity in psychosis is associated with brain expression of schizophrenia-related genes. *Proc Natl Acad Sci U S A* 116:9604-9609.

24. Xia MR, Wang JH, He Y (2013): BrainNet Viewer: A Network Visualization Tool for Human Brain Connectomics. *PLoS One* 8.

25. Cline MS, Smoot M, Cerami E, Kuchinsky A, Landys N, Workman C, et al. (2007): Integration of biological networks and gene expression data using Cytoscape. *Nat Protoc* 2:2366-2382.
